# Towards a comprehensive evaluation of dimension reduction methods for transcriptomic data visualization

**DOI:** 10.1038/s42003-022-03628-x

**Published:** 2022-07-19

**Authors:** Haiyang Huang, Yingfan Wang, Cynthia Rudin, Edward P. Browne

**Affiliations:** 1 Department of Computer Science, Duke University, Durham, NC 27708 USA; 2 Department of Medicine, University of North Carolina at Chapel Hill, Chapel Hill, NC 27599 USA

**Keywords:** Machine learning, Data mining, Data processing

## Abstract

Dimension reduction (DR) algorithms project data from high dimensions to lower dimensions to enable visualization of interesting high-dimensional structure. DR algorithms are widely used for analysis of single-cell transcriptomic data. Despite widespread use of DR algorithms such as t-SNE and UMAP, these algorithms have characteristics that lead to lack of trust: they do not preserve important aspects of high-dimensional structure and are sensitive to arbitrary user choices. Given the importance of gaining insights from DR, DR methods should be evaluated carefully before trusting their results. In this paper, we introduce and perform a systematic evaluation of popular DR methods, including t-SNE, art-SNE, UMAP, PaCMAP, TriMap and ForceAtlas2. Our evaluation considers five components: preservation of local structure, preservation of global structure, sensitivity to parameter choices, sensitivity to preprocessing choices, and computational efficiency. This evaluation can help us to choose DR tools that align with the scientific goals of the user.

## Abstract (web summary)

The authors provide an evaluation framework for dimension reduction methods that illuminates the strengths and weaknesses of different algorithms, and applies this framework to evaluate the PCA, t-SNE, UMAP, TriMap, PaCMAP, ForceAtlas2, and PHATE algorithms.

## Introduction

Dimension reduction (DR) algorithms have emerged as critical tools that allow scientists to gain insight into high-dimensional data. DR algorithms map high-dimensional data to a low-dimensional embedding, enabling data visualization. A high-quality visualization can help the user to gain insights about cluster structure and distributional characteristics of the data. On the other hand, a low-quality DR visualization can create the appearance of structure in the data that does not actually exist. For instance, DR methods often produce false clusters, where subgroups of data cluster separately despite few, if any, underlying biological differences. Unreliable discoveries from DR results can lead to misguided and wasted effort trying to interpret and clarify false clusters. Furthermore, DR methods can be highly sensitive to the parameter and pre-processing choices, so that seemingly innocuous choices by users can completely dismantle the true structure of the data, leaving the user without a way to explore the data or generate useful hypotheses.

**Fig. 1:**
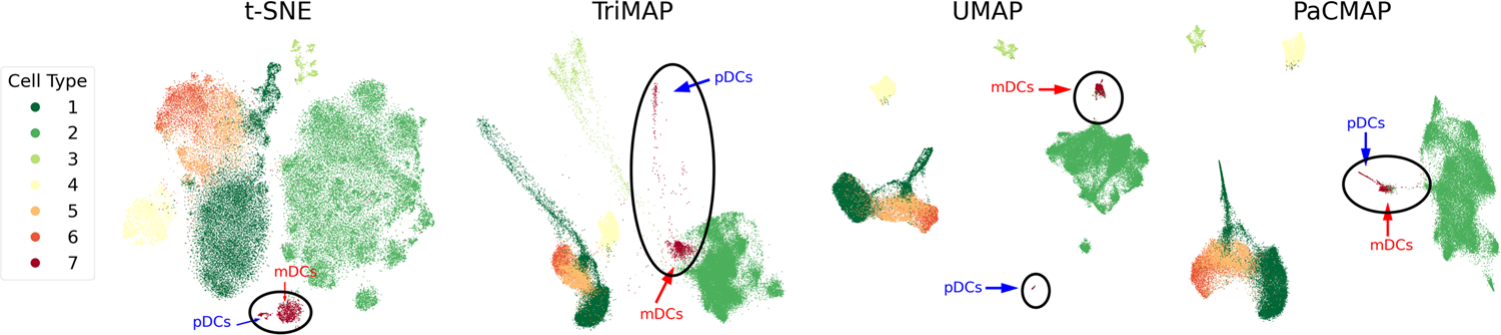
Comparison of global structure preservation for DR methods. DR visualizations of a scRNAseq dataset of PBMCs from HIV-infected people from ref. ^6^, pre-processed using the top 70 principal components. Labels for cell types were identified by differential expression analysis for each cluster. DC clusters are circled. Note that DR algorithms can behave differently under different random seeds, and we discuss the implication of such behavior in Supplementary Note 3 and Supplementary Fig. 5.

DR is used extensively in the analysis of single-cell transcriptomics data^1–3^ and other types of high-dimensional cytometry^4,5^ (for a detailed evolution of the dimensionality reduction method for transcriptomics data, please see Supplementary Note 1). These are data types for which DR methods have the potential to pinpoint clusters that are biologically meaningful, but also have the potential for false clusters that could be misinterpreted as interesting biological entities. To demonstrate these points by directly comparing DR methods, we ran parallel DR analyses using a benchmark dataset. Figure 1 shows DR visualizations of single-cell RNA sequencing (scRNA-seq) data derived from peripheral blood mononuclear cells (PBMCs) from HIV-infected individuals^6^. Altogether, the data include transcriptional profiles from 59,286 cells. The clusters are annotated by comparing differentially expressed genes defining each cluster to known lineage markers and previously published datasets. The dataset was processed by four DR algorithms, which are t-SNE with the FIt-SNE implementation^7–9^ (denoted as t-SNE), UMAP^10^, TriMap^11^, and PaCMAP^12^. PaCMAP is a recent method that is designed to optimize both global and local structure. In the data, dendritic cells (DCs) are present in blood as two major subsets—myeloid DCs (mDCs) and plasmacytoid DCs (pDCs) defined by expression of CD1c or CD303, respectively. However, in the results shown in Fig. 1, UMAP separates the two clusters of dendritic cells (mDCs and pDCs) into two spatially distant groups, whereas t-SNE, TriMap, and PaCMAP correctly map these DC subsets close to each other.

In the cases in Fig. 1, prior knowledge of DC subtypes and blood cell progenitors allows us to identify the most accurate embedding but, in other cases, ground truth labeling may not exist, forcing us to rely on the DR results. Another example is shown in Supplementary Fig. 1, showing cases where local or global structure in scRNA-seq data is not well preserved in visualizations.

DR methods often become widely used without being carefully evaluated, and these methods may contain flaws that are unknown to their users. As further evidence of this, there are now many papers explaining how to use various popular DR methods effectively e.g., “The Art of t-SNE,”^13^ and “How to use t-SNE effectively”^14^. These papers are only necessary because DR results are often misleading, and because DR cannot be trusted out-of-the box^15,16^. These papers, which teach us how to manipulate parameters of DR algorithms, highlight the urgent need to develop trustworthy DR methods. For this to be achieved, we will first need to establish benchmarks regarding how DR evaluations could be conducted, which is the purpose of this work.

Here, we propose an evaluation framework for DR methods in biological domains. This framework includes many different essential elements of DR results, such as local structure preservation, global structure preservation, sensitivity to parameter choices, sensitivity to pre-processing choices, computational efficiency, and scalability. Many of these metrics are used ad hoc in other papers but have not previously been integrated into a comprehensive evaluation approach.

To demonstrate our proposed evaluation framework, we compared eight DR methods. The eight methods are PCA^17^, t-SNE with the FIt-SNE variant (implemented by openTSNE^7–9^ and denoted as t-SNE), t-SNE with the hyperparameter described by Kobak and Berens^13^ (denoted as art-SNE, see Supplementary Note 4.1 for the reason for the difference between two t-SNE variants), UMAP^10^, TriMap^11^, PaCMAP^12^, ForceAtlas2^18,19^, and PHATE^20^.

By applying this evaluation framework to popular DR algorithms, we confirm findings from past works that t-SNE^7^ and UMAP^10^ are highly sensitive to the parameter and/or pre-processing choices and do not perform well with respect to global structure metrics^12,13,15^. Furthermore, we find that the recent PaCMAP method^12^ performs well in comparison to previous methods. Nevertheless, our analyses indicate there is still substantial room for improvement in DR methodology.

Our framework is built upon the intuition that, ideally, a DR method would preserve local structure and global structure, be somewhat insensitive to parameter choices and pre-processing and be computationally efficient. In this work, we propose evaluation criteria for each of these qualities, and use these criteria to evaluate the performance of several popular DR methods using publicly available datasets. Here, local structure preservation means that neighbors in the high-dimensional space should still be neighbors in the low-dimensional space. More generally, local structure is preserved when local neighborhoods in the high-dimensional space are similar to local neighborhoods in the low-dimensional space. Global structure preservation means that relative positions between clusters are preserved, as well as larger-scale manifold structures.

Based on our experiments, t-SNE, art-SNE, UMAP, and PaCMAP perform well on local structure preservation metrics with t-SNE and art-SNE performing the best on an unsupervised metric, while PCA, TriMap, PaCMAP, and ForceAtlas2 perform well on global structure preservation metrics and are robust to parameter choices. PaCMAP is robust to pre-processing choices and achieves the lowest running time.

## Results

### Evaluation 1: local structure preservation

We consider two local evaluation methods as part of our framework: Local Supervised Evaluation and Local Unsupervised Evaluation.

Local supervised evaluation requires a labeled classification dataset, in the form (**x**_*i*_, *y*_*i*_), for *i* = 1, …, *n*, where **x**_*i*_ is a high-dimensional vector and *y*_*i*_ takes a value between 1 and *M*, where there are *M* classes.

To perform local structure evaluation, we perform DR on the **x**_*i*_’s, associate each *i* with its label *y*_*i*_, run a supervised classification algorithm on a subset of the low-dimensional data, and report classification accuracy on the rest of the low-dimensional data.

We typically choose support vector machines (SVM) with radial basis function kernels following ref. ^12^ or *k*-nearest-neighbor (*k*NN, *k* = 5) classifiers following ref. ^10^ as our classifiers because they are flexible and nonparametric.

The principle that this evaluation is premised on homophily: members of each class should be close to other members of the same class, and should be far from members of other classes. Then, when we project the data using DR, the same property should hold.

**Fig. 2:**
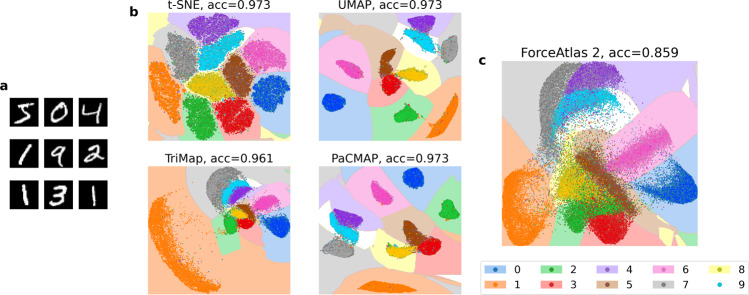
Measuring local structure preservation with SVM supervised classification. **a** Nine samples out of the 70,000 total samples from the MNIST dataset. **b** DR results from four different methods using the MNIST dataset. acc indicates test accuracy score. The accuracy score and decision boundaries were created by an SVM classifier. Here, 90% of the data were used for training, 10% for testing. **c** Results of ForceAtlas2 on the MNIST dataset, following the same procedures. ForceAtlas2 demonstrates inferior local structure preservation compared to the other DR methods.

Figure 2 and Supplementary Fig. 3 provide evaluations of local structure preservation for a complex high-dimensional dataset–the MNIST handwritten digit dataset^21^. An important property of this dataset is that the major clusters (representing each digit) are all well separated in high-dimensional space. Most DR methods (t-SNE, UMAP, TriMap, and PaCMAP) are able to preserve this structure when projecting to 2-D, based on evaluation with SVM and *k*NN (*k* = 5) (Fig. 2 and Supplementary Fig. 3). By contrast, ForceAtlas2 performs poorly and fails preserve local structure well (Fig. 2c).

**Fig. 3:**
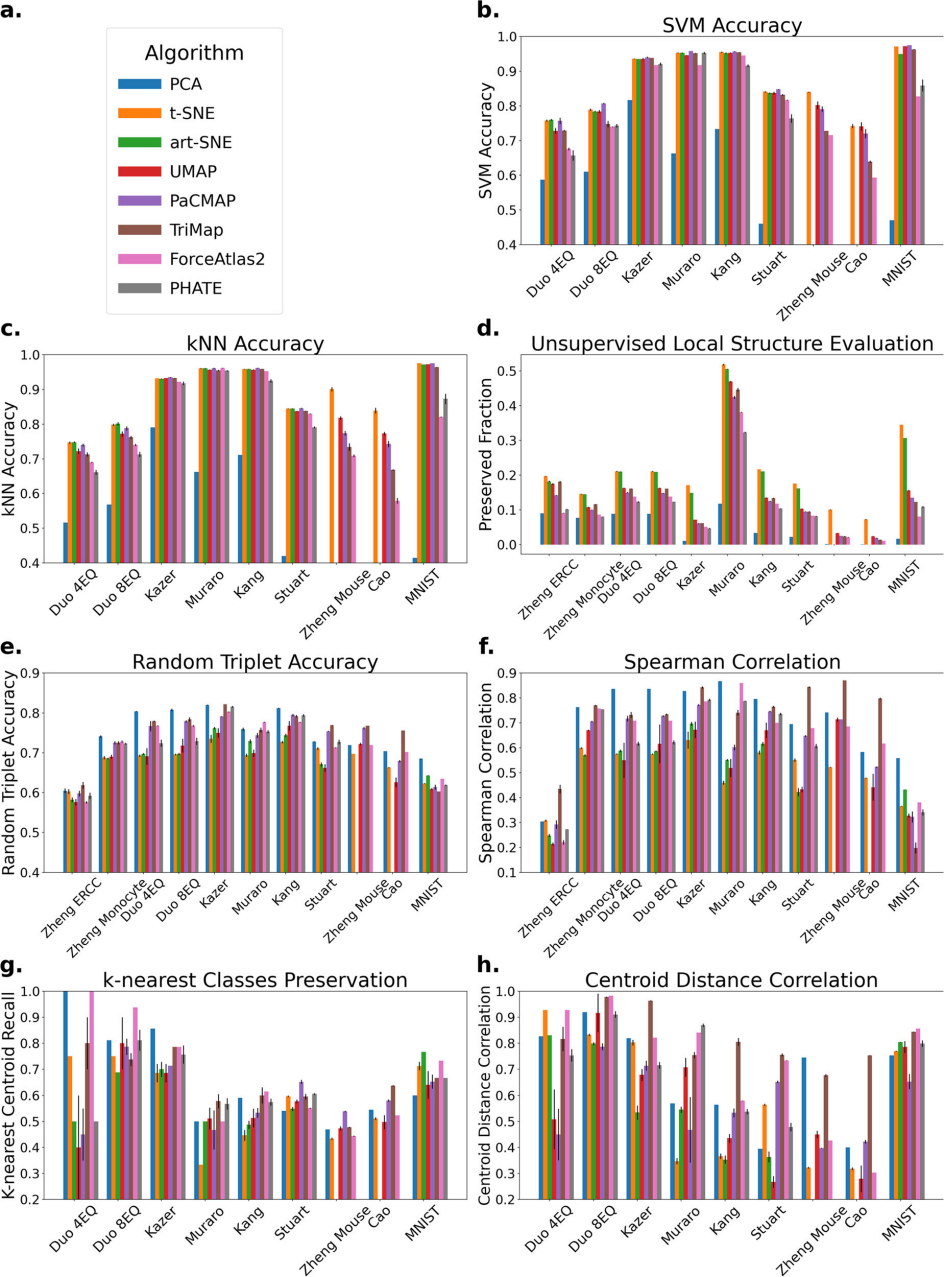
Local and global structure evaluations. Bar plots represent the average evaluation metrics across five runs for each algorithm with standard deviation shown as black error bars. Results of art-SNE and PHATE on Zheng Mouse and Cao are not included in this figure since they are unable to finish these large datasets under a time/memory budget—see the caption of Supplementary Table 4 for more information. Zheng ERCC and Zheng Monocyte are only used for unsupervised evaluation metrics. **a** Legend for all subfigures. **b** Local structure (supervised): SVM evaluation. Here methods that focus on local structure, such as t-SNE, art-SNE, UMAP, and PaCMAP, perform well. **c** Local structure: kNN evaluation. Similar to (**b**), t-SNE, art-SNE, UMAP, and PaCMAP perform well on this metric. **d** Local structure: proportion of neighborhood preserved. t-SNE and art-SNE achieved the best neighborhood preservation ability on the datasets. **e** Global structure: random triplet accuracy. TriMap, PaCMAP, and ForceAtlas2 perform well on this evaluation metric. **f** Global structure: distance Spearman correlation. Consistent with (**e**), here PCA, TriMap, PaCMAP and ForceAtlas2 perform well. **g** Global structure: k-nearest classes preservation. ForceAtlas2 and PCA perform the best on this metric. **h** Global structure: centroid distance correlation. On this metric, TriMap, and ForceAtlas2 perform well.

The prediction accuracy scores of the SVM and *k*NN (*k* = 5) classifiers over multiple datasets and DR methods are reported in Supplementary Table 1 and Supplementary Table 2 and summarized in Fig. 3b, c. Note that the results of art-SNE and PHATE on Zheng Mouse and Cao are not included in the figure since they are unable to finish these large datasets under a time budget—see the caption of Supplementary Table 4 for more information. All methods except ForceAtlas2 perform well on SVM evaluation, with t-SNE, art-SNE, UMAP, and PaCMAP arguably achieving the best results. On *k*NN evaluation, t-SNE, art-SNE, UMAP, and PaCMAP achieve the best results, while ForceAtlas2 and PHATE generate comparatively poor results.

Next, we propose another local evaluation method, this time with unsupervised data (no *y*_*i*_’s are available). In many cases, ground truth labels are not known, such as when analyzing scRNA-seq or flow cytometry data. In this case, the quality of the visualization is evaluated based on how accurately the neighborhood information from high dimensions has been preserved by each algorithm.

To perform this evaluation, we compute **N**(*i*), the set of *k* = 5 nearest neighbors for each point *i* in the high-dimensional space, and ${{{{{{{\bf{N}}}}}}}}^{\prime} (i)$, the *k*-nearest neighbors in the low-dimensional space. We compute the proportion of intersection between **N**(*i*) and ${{{{{{{\bf{N}}}}}}}}^{\prime} (i)$, and then compute the average proportion of neighbors preserved over all *i*.

This evaluation over multiple datasets and DR methods is reported in Supplementary Table 3 and Supplementary Note 5.2, and summarized in Fig. 3d. Using this approach, t-SNE and art-SNE exhibited performance superior to the other DR methods by achieving the highest fraction of neighborhood structure preserved on all datasets selected. By contrast, PCA achieved relatively poor results.

### Evaluation 2: global structure preservation

Unlike local structure, which focuses on the relationships between points in a neighborhood, global structure focuses on relationships between neighborhoods. A DR result with high-quality global structure preservation could help us understand, for instance, the degree of similarity between different groups of cells.

**Fig. 4:**
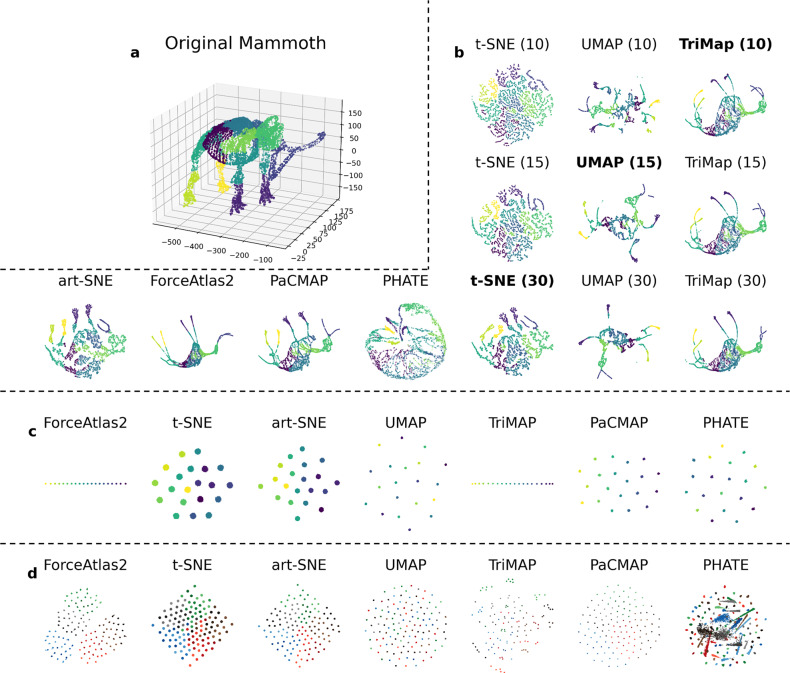
Qualitative evaluation of global structure preservation using the Mammoth dataset, the Gaussian Linear dataset, and the Gaussian Hierarchical dataset. **a** Visualization of the original Mammoth dataset. **b** DR results on the Mammoth dataset. Results of art-SNE, ForceAtlas2, PaCMAP and PHATE are created under default configurations (these algorithms automate their own parameter tuning), and results of DR algorithms t-SNE, UMAP, and TriMap are created under different parameter configurations. We tune the most important parameter that controls the visual presentation of the figure, which is perplexity for t-SNE, n_neighbors for UMAP and n_inliers for TriMap, with the parameter specified in the brackets. All DR algorithms preserve local structure well, while only TriMap, PaCMAP, and ForceAtlas2 preserve global structure well. **c** Results of DR algorithms on the Gaussian Linear dataset. Sample points are annotated in gradient color, demonstrating their relative location in the high dimensional space. Although t-SNE, PHATE and UMAP successfully preserve the local structure, the global structure is completely lost and the gradual change of color observed in other visualizations is not observed. **d** Results of DR algorithms on the Hierarchical Gaussian dataset. The Hierarchical Gaussian dataset is particularly difficult, because it contains structure at multiple scales that needs to be preserved. Here the points are colored by their meso-level cluster assignment (see definition in Supplementary Note 7.1). t-SNE, UMAP and TriMap do not perform well, since micro clusters from the same meso clusters are not placed together, whereas art-SNE and PacMAP perform better and ForceAtlas2 performs quite well. PHATE fails to preserve local structure on this dataset.

An algorithm with high-quality global structure preservation would, for example, preserve separate clusters of the major cell lineages within PBMCs, and be less likely to create false clusters or to separate a single cluster into two far away subclusters. Figure 4a, b illustrates DR results on the Mammoth dataset^22,23^, a 3-D dataset that we project to 2-D (think of crushing the mammoth onto the page like a leaf). This is a dataset for which global structure preservation is particularly important, and ideally, DR will generate a 2-D picture that preserves recognizable mammoth characteristics. Using this dataset, we observed that the performance of several popular DR methods was poor, in that they separate connected parts of the mammoth into false clusters. t-SNE and UMAP in particular generate results that bare little resemblance to the original mammoth.

We propose qualitative and quantitative evaluations for determining the performance of DR methods in preserving global structure. Both approaches are considered below.

The evaluation of the mammoth dataset above is an example of a qualitative evaluation—we know the mammoth’s true 3-D structure, and we know that the 2-D projections do not fully reflect it.

Intuition can also be helpful when we have some biological knowledge about the relationships between parts of the plot; e.g., that certain cell types are related to each other as in Supplementary Fig. 2, and thus should be close to each other in the DR projection.

Simulated data examples can also be very helpful here to qualitatively evaluate DR preservation of the global structure. We give two examples with novel simulated datasets with different properties in Fig. 4c, d: a Gaussian Linear dataset and a Gaussian Hierarchical dataset. Detailed generation procedures for the Gaussian Hierarchical dataset can be found in Supplementary Note 7.1.

In Fig. 4c, we observe from DR projections of these datasets that, although most DR algorithms successfully capture the general arrangement of the clusters, t-SNE and UMAP perform poorly at preserving this global structure. In this dataset, the sample points are annotated in gradient color that demonstrates their relative location in high-dimensional space. Nevertheless, the gradual change of color that can be observed in the embedding of ForceAtlas2, TriMAP, and PaCMAP cannot be observed in this embedding for t-SNE, art-SNE, UMAP, and PHATE. TriMAP and ForceAtlas2 successfully preserve the line structure of the dataset, whereas the other algorithms make use of limited visual space instead by spreading their clusters out. In Fig. 4(d), we observe that PHATE fails to preserve the local structure. t-SNE, UMAP, and TriMAP, while preserving the local clusters, fail to preserve the hierarchical structure. art-SNE, PaCMAP, TriMAP, and ForceAtlas2 partially retain this information, since the micro-level clusters from the same meso-level clusters are placed closer to each other. However, ForceAtlas2 fails to separate meso-level clusters from each other; the boundary between different meso/macro clusters would be unclear without coloring according to the labels. The other methods additionally fail to separate both meso-level and macro-level clusters.

Besides qualitatively evaluating global structure, we also provide quantitative analysis. We first analyze the preservation of distances between sample points. For this objective, we recommend use of a quantitative evaluation metric called random triplet accuracy first used in ref. ^12^, which is the percentage of triplets (all combinations of three data points) whose relative distance in the high- and low-dimensional spaces maintain their relative order. For numerical tractability, we use a sample of triplets rather than considering all triplets.

The random triplet accuracy evaluation over multiple datasets and DR methods is reported in Supplementary Table 6 and summarized in Fig. 3e. Notably, TriMap exhibits superior (higher) triplet accuracy than other approaches, possibly because TriMap optimizes a triplet loss. TriMap also tends to perform well on other qualitative global structure assessments when compared to other DR methods. PCA, PaCMAP and ForceAtlas2 also perform well in this evaluation.

Based on similar ideas, we evaluate the distance Spearman correlation, which is the Spearman rank-order correlation between two vectors: distances between pairs of points in the high-dimensional space and distances between those same points in the low-dimensional space. Again, due to numerical tractability, we use a sample of pairs rather than considering all pairs in the dataset. The distance Spearman correlation evaluation over multiple datasets and DR methods is reported in Supplementary Table 7 and summarized in Fig. 3f. In this evaluation, PCA, PaCMAP, TriMap, and ForceAtlas2 perform well on most datasets, while t-SNE, art-SNE, and UMAP perform relatively worse.

Beyond evaluating the preservation of relationships between sample points, we also look into another perspective: the preservation of relationships between different clusters. Evaluation under this perspective, however, may not be as accurate as those that assess relationships between different sample points, due to label inaccuracies and class imbalance that widely exist in scRNA-seq datasets. We use two metrics to measure the preservation of cluster relationships based on the relative location of the centroids. The first one is the *k*-nearest class preservation, first introduced in Kobak and Berens^13^. This metric evaluates the fraction of the *k*-nearest-neighbor classes that are preserved in the low-dimensional embedding. The neighboring relationship is defined by the relative distances between cluster centroids. The number of classes in the datasets we selected varies a lot, and choosing the same *k* across datasets could ambiguate the meaning of this metric. Therefore, we choose *k* to be a dynamic value, $k=\left\lfloor \frac{C+2}{4}\right\rfloor$, where *C* is the number of classes in the dataset. This evaluation over multiple datasets and DR methods is reported in Supplementary Table 8 and summarized in Fig. 3g. In this evaluation, every algorithm performs well on some datasets and performs poorly on other datasets. In general, PCA and ForceAtlas2 have better performance across datasets.

The third metric is the centroid distance correlation, which evaluates the Spearman correlation between the set of centroid distances in the high-dimensional space, and the set of distances between centroids in the low-dimensional space. This evaluation over multiple datasets and DR methods is reported in Supplementary Table 9 and summarized in Fig. 3h. Similarly, TriMap and ForceAtlas2 have better performance across all datasets by achieving relatively high centroid distance correlation score on most datasets. Note that some algorithms perform well on some datasets while performing poorly on others, for example, t-SNE performs well on Duo 4Eq^24^ but performs poorly on Muraro^25^ and Kang^26^.

### Evaluation 3: sensitivity to parameter choices

An ideal DR algorithm should avoid complicated and confusing parameter tuning. Ideally, one set of parameters works for most datasets. The volatility of DR outcome during parameter tuning raises doubts about the reliability of the results. How should we tune these parameters? Without ground truth labels, tuning requires qualitative evaluation of the visualization in light of some of the known facts, but, in doing this, we risk the introduction of human bias into parameter selection (for a detailed discussion of parameter choices, please see Supplementary Note 9).

**Fig. 5:**
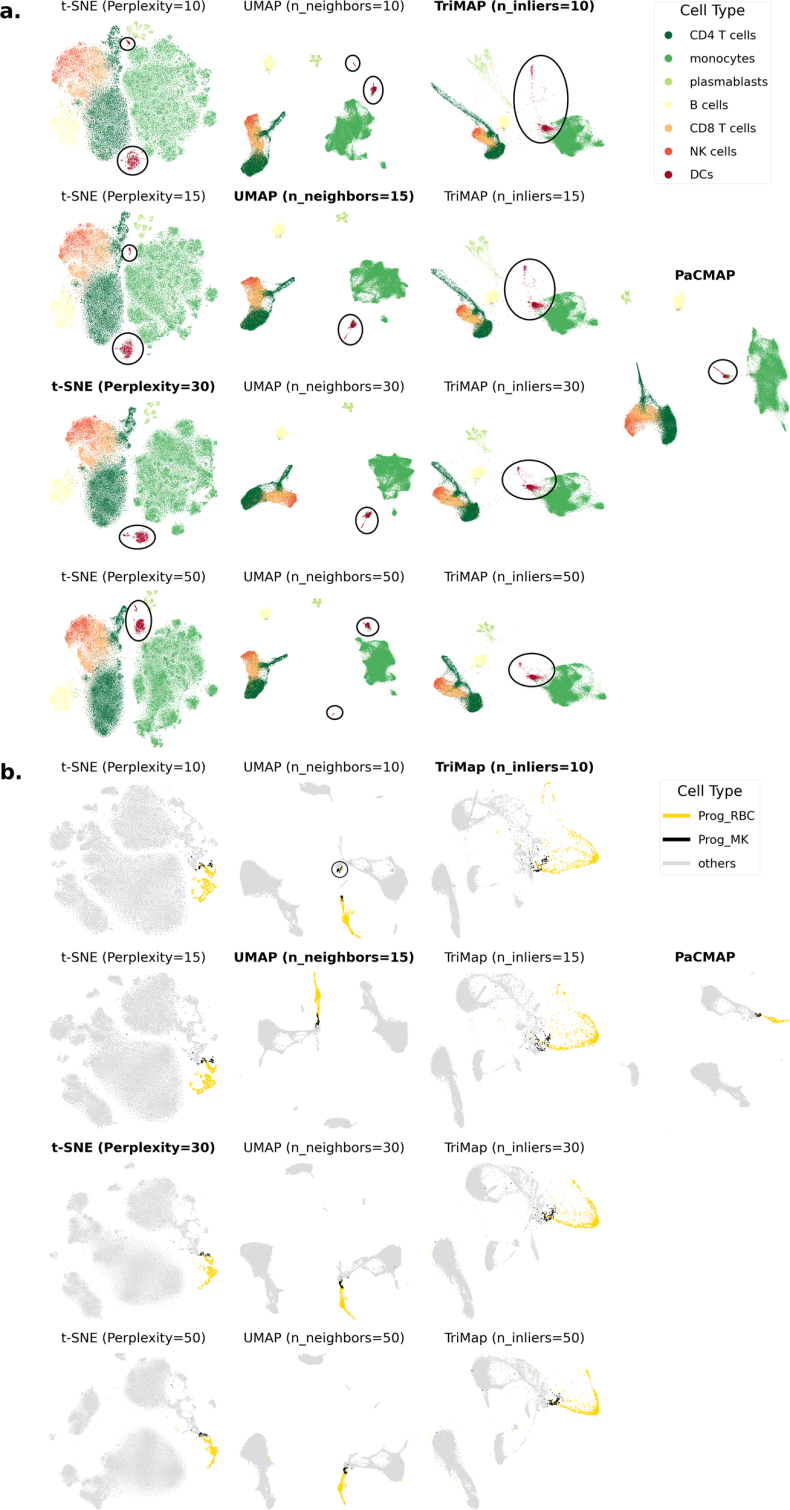
Sensitivity to parameter choices on the Kazer et al.^6^ dataset and Stuart et al.^30^ dataset. **a** For the Kazer et al.^6^ dataset, the distance relationship between DC clusters varies dramatically when tuning algorithm parameters in t-SNE and UMAP. The parameter we varied in each algorithm controls the spread of its attractive forces around each point. Clusters of DCs are circled, showing that in some cases, a single-cell type was split into two clusters by DR. The dataset was pre-processed by PCA with 70 PCs generated. **b** For the Stuart et al.^30^ dataset, the distance relationship between red blood cell progenitors (Prog_RBC) and megakaryocyte progenitors (Prog_MK) varies dramatically when tuning algorithm parameters in UMAP. Again, the parameters we varied control the spread of attractive forces. The dataset was pre-processed by PCA with 70 PCs generated.

Figure 5a provides an example of how parameter tuning can change DR results. In this figure, the distance between the two clusters of DCs varies with different parameter choices for t-SNE and UMAP. In addition, in the t-SNE results, the distance between clusters of plasmablasts and B cells varies substantially depending on parameter choices. Figure 5b provides another example, where the distance between red blood cell progenitors (“Prog_RBC”) and megakaryocyte progenitors (“Prog_MK”) varies considerably with different parameter choices for UMAP. This demonstrates that these algorithms are sensitive to parameter choices, which is not ideal.

### Evaluation 4: sensitivity to pre-processing choices

**Fig. 6:**
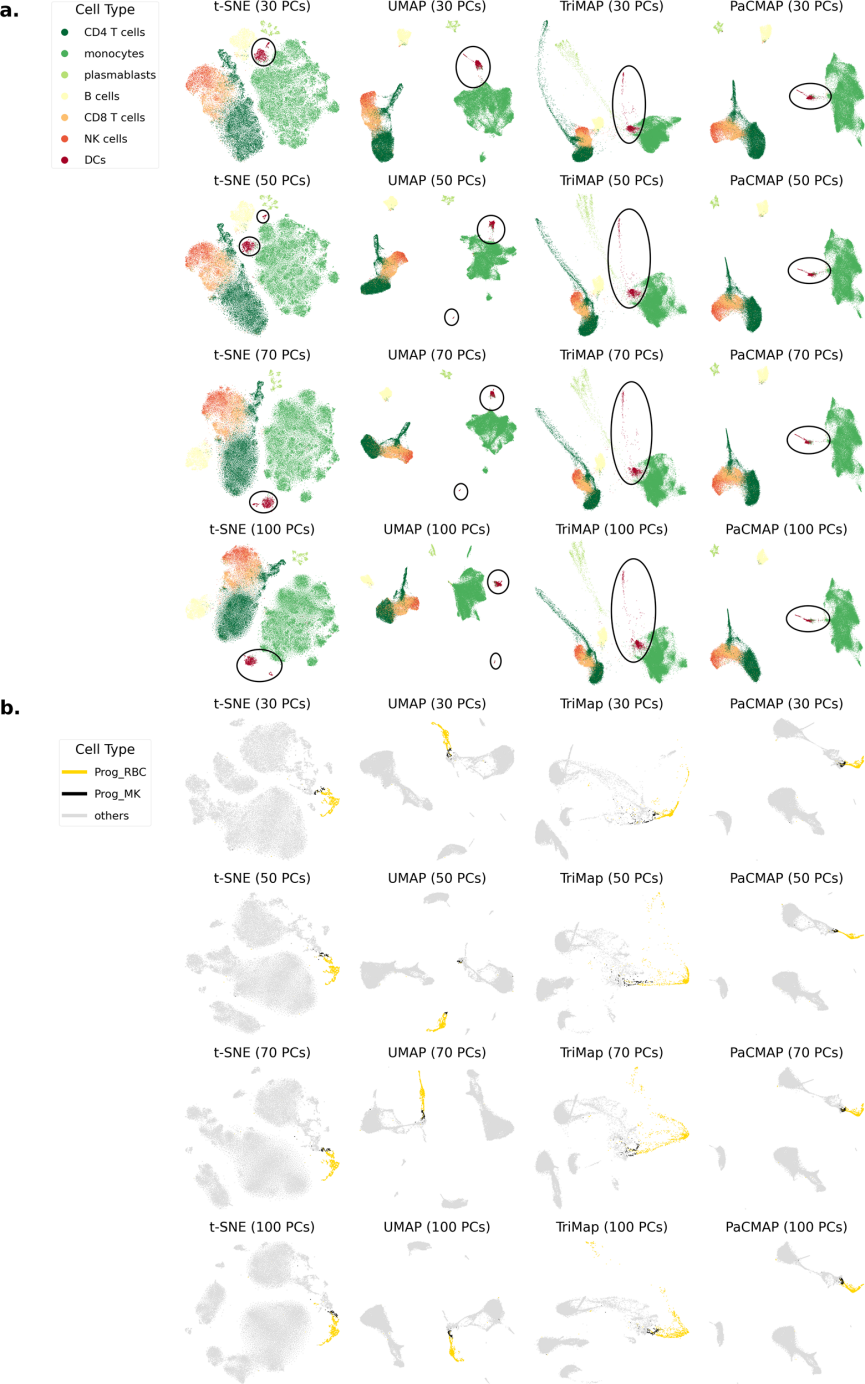
Sensitivity to the number of PCA dimensions used to initialize DR. **a** Here we varied the number of PCs on the Kazer et al.^6^ dataset. t-SNE and UMAP were not robust to the number of PCs from PCA pre-processing, having different distance relationships between two DCs clusters (pDCs and mDCs), while TriMap and PaCMAP are relatively robust in this respect. **b** We varied the numbers of input PCs from the Stuart et al.^30^ dataset. Here, UMAP is not robust to the number of PCs from PCA pre-processing, generating different results in terms of distance between red blood cell progenitors (Prog_RBC) and megakaryocyte progenitors (Prog_MK) depending on the number of PCs, while t-SNE, TriMap and PaCMAP are rather robust in this aspect. For this dataset, cell types Prog_RBC and Prog_MK should be close to each other, but UMAP with 50 PCs places points belonging to these two cell types far away from each other. Additionally, the Prog_MK cluster is separated into two clusters in UMAP's result with 50 PCs.

An ideal DR method should be robust to pre-processing methods. Conventional pre-processing steps, as defined in ref. ^27^, consist of log-normalization and PCA. PCA is often used to reduce the number of dimensions to below 100 (in some cases, to below 20) before applying DR. Using PCA after log-normalization has two advantages: it reduces the computational time for DR, and it tends to improve the stability of DR. However, PCA pre-processing often does not preserve the original distances in the high-dimensional space and could potentially harm the overall results. Ideally, DR methods should be somewhat insensitive to the number of principal components (PCs) from PCA pre-processing that are used. Figure 6 shows DR results from two RNAseq datasets, showing that t-SNE and UMAP’s results in terms of distance for related cell types (e.g., mDCs and pDCs) are not generally robust to the number of PCs chosen, while TriMAP and PaCMAP are relatively robust.

**Fig. 7:**
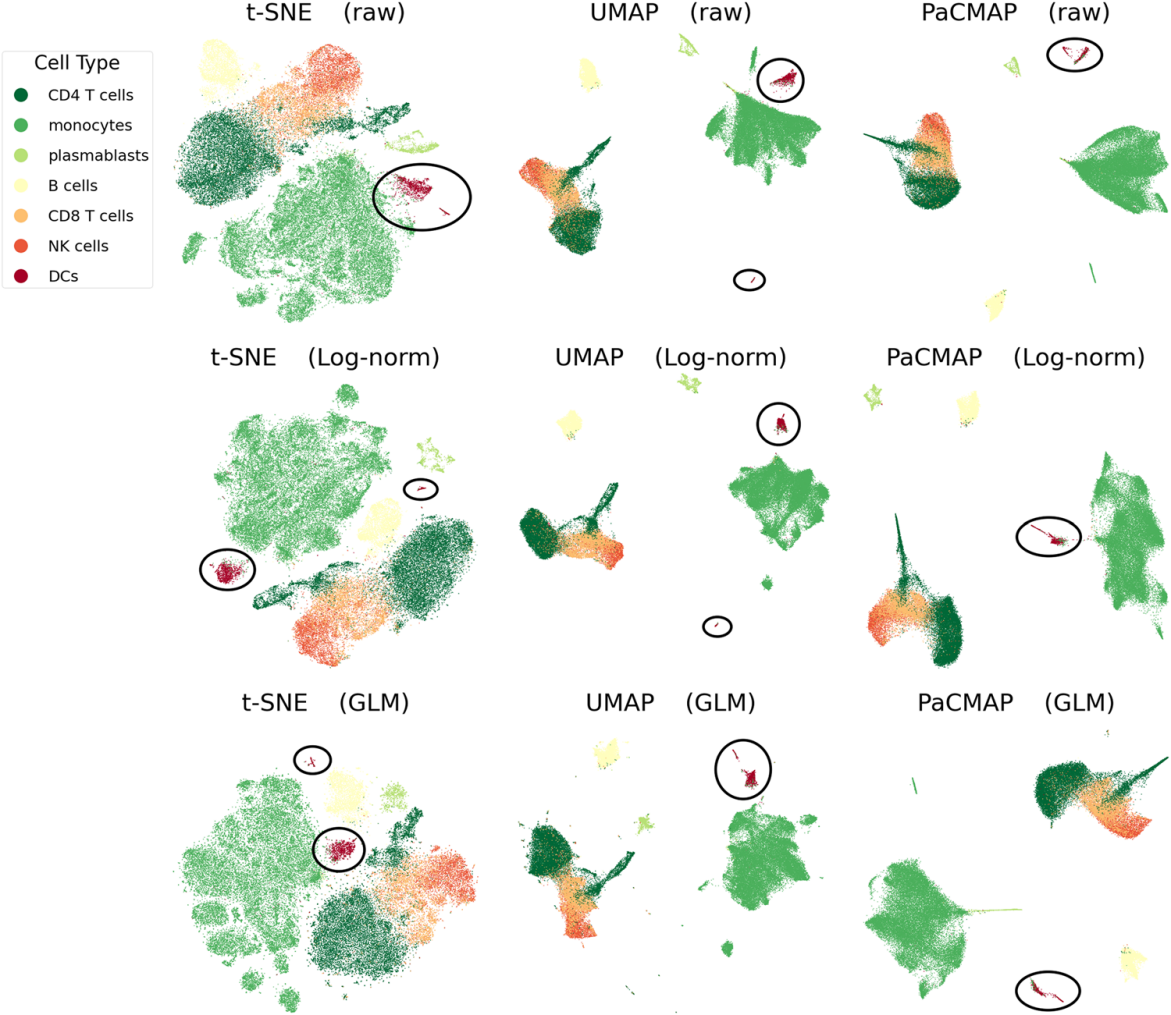
Sensitivity of DR results to pre-processing methods on the Stuart et al.^30^ dataset. This dataset was pre-processed either with either no PCA and no log transformation (raw), log-normalization (Log-norm) or by GLM-PCA. The distance relationship between two of the DCs clusters (mDCs and pDCs) varies dramatically in t-SNE and UMAP when changing pre-processing methods. There are unfavorable outliers and tiny clusters in t-SNE and UMAP results with GLM-PCA pre-processing. For raw data and log-normalized data, the number of dimensions from PCA is 70, for GLM-PCA pre-processed data, the number of PCA dimensions is 50. GLM-PCA is computationally infeasible to run for more than 50 PCA dimensions.

Other commonly used pre-processing methods for scRNAseq data, such as log-normalization, could also impact the outcome of the DR tools. In an experiment, we selected the following three pre-processing step combinations: (1) no log-normalization (raw data) and include PCA, (2) include log-normalization and include PCA, (3) include log-normalization and use the GLM-PCA method of ref. ^28^. The experimental results are shown in Fig. 7. This figure shows that t-SNE and UMAP are not robust to pre-processing methods, and the distance relationship between two DCs clusters (mDCs and pDCs) varies dramatically when changing the pre-processing methods. By contrast, PaCMAP was found to be relatively robust to these pre-processing choices. t-SNE and UMAP’s results using GLM-PCA pre-processing also produced a large number of outliers and tiny clusters compared to PaCMAP.

### Evaluation 5: computational efficiency and scalability

DR algorithms are known for their long-running times when handling large, high-dimensional datasets. This is problematic since with the advancement of transcriptomic technology, scRNA-seq datasets are increasing in size, from hundreds of cells per sample to tens of thousands of cells per sample. A faster algorithm could save time for further analysis and allow researchers to try different parameters to discover underlying structure more efficiently.

**Fig. 8:**
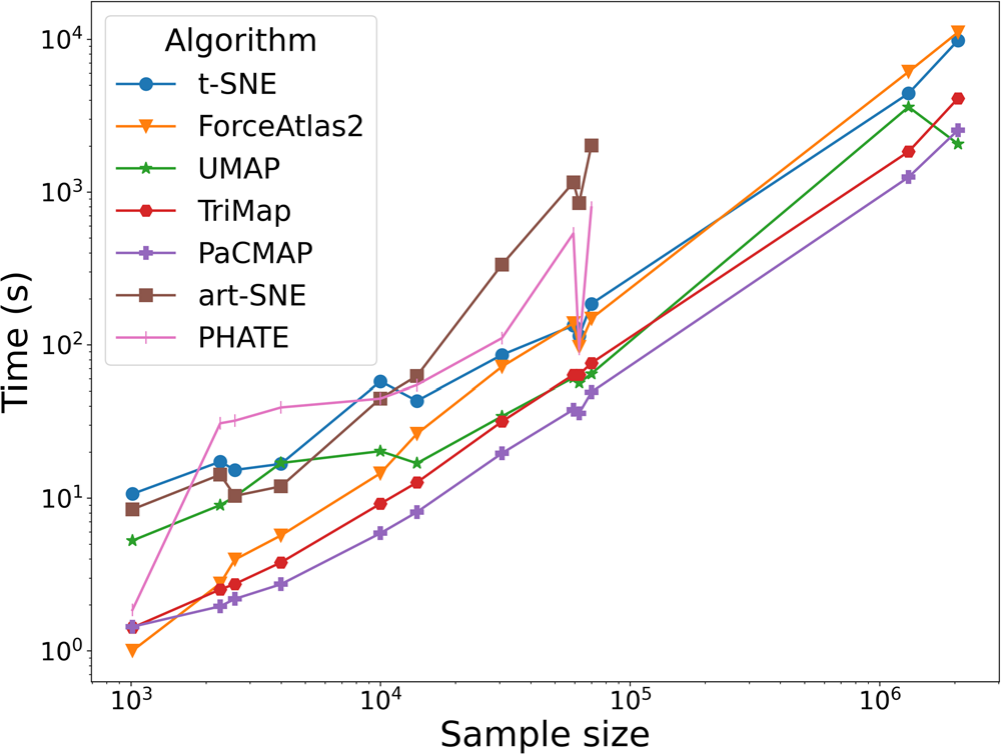
Running time comparison. For each algorithm, the running time is defined as the time required to transform the pre-processed dataset with shape *N* × *d* into a low-dimensional embedding with shape *N* × 2, using the default convergence criteria for each algorithm. Therefore, for ForceAtlas2, the nearest-neighbor graph construction time is also included for fairness. For the Zheng Mouse^24^ (sample size: 1306127) and Cao^3^ (sample size: 2058652) datasets, art-SNE ran out of memory, and PHATE cannot finish these datasets within a time limit of 24 h.

Figure 8 summarizes the running times of different DR algorithms over the datasets with different sample size discussed in this paper. More details of running times can be found in Supplementary Table 4. Consistent with our previous work, Wang et al.^12^, PaCMAP achieves the fastest run time for most of the datasets, especially for large-scale datasets such as Zheng Mouse^24^, and PaCMAP and UMAP achieve comparable results on datasets with sample size over 10000. UMAP, TriMap, and t-SNE (with its recent implementations) can also complete dimensionality reduction for these datasets in reasonably short runtimes, while ForceAtlas2, PHATE, and art-SNE are much slower. Indeed, art-SNE, as anticipated by Kobak and Berens^13^, was unable to process these datasets under the given memory limit. PHATE was unable to process the large-scale datasets within a given budget of time (24 h).

## Discussion

In this work, we propose five different ways to evaluate DR algorithms. By demonstrating this thorough evaluation on current DR methods, our analysis yielded insight into their strengths and weaknesses with various datasets. Several of the widely used DR methods, such as t-SNE and UMAP have difficulty capturing global structure, which leads to the possibility of misinterpreting “false” clusters as real clusters, resulting in false hypothesis generation. In agreement with ref. ^29^, we found that the algorithms that preserve local structure not only tended to perform poorly on global structure metrics, but also tended to perform poorly on robustness and sensitivity checks, frequently leaving the results as an arbitrary byproduct of choices made within the algorithm. Finally, some DR methods are substantially more efficient than others and can handle much larger datasets, which is an important practical consideration, particularly for conducting some of the other evaluations that require repeated runs of an algorithm. We also observed that run time does not necessarily correlate with either the quality of local or global structure preservation. Another observation we made, through the use of simulated datasets, is that even the most reliable DR methods often have trouble preserving hierarchical (or more intricate) types of structures.

The framework we present here will be helpful for practitioners and designers of DR methods. Importantly,

- Random triplet loss is a useful gauge of global structure preservation, which should correlate with robustness and stability to pre-processing choices. By examining triplet loss (which can be done with either labeled or unlabeled data), one can gain a sense of whether global structure might be preserved in a DR projection of the data. Our results show that t-SNE, UMAP, and several other methods do not perform well with respect to this metric, but that others such as TriMap and PaCMAP do perform well.
- Since robustness is correlated with global structure preservation, if one desires the DR results to be more robust to parameter choices, then it is worthwhile to choose an algorithm designed for global preservation, and to perform the evaluation in the section “Evaluation 3: sensitivity to parameter choices”. Our results show that t-SNE and UMAP are not usually robust; they provide results that vary under different parameter selections within the reasonable range.
- If the dataset is large, the user should consider algorithms that have better scalability according to Supplementary Table 4. Scalability is important especially when one would like to choose different parameters for investigation and thus needs to run the algorithm repeatedly.
- If the user does not have knowledge about whether local structure or global structure is important in the dataset, an algorithm that performs well on both local- and global- structure preservation could be helpful, according to Supplementary Table 1, Supplementary Table 2, and Supplementary Table 6.

In writing this paper, we envisioned the user would evaluate their chosen DR methods on several datasets besides their own to understand whether the results on their dataset might be trustworthy in specific ways: does the algorithm tend to create false clusters? Is it sensitive to initial conditions? The answers to these questions inform how trustworthy the DR results are and whether one should consider acting on what is shown in the DR projections.

## Methods

For all the methods except ForceAtlas2, we used their default hyperparameter setting. Due to its diffusion-based nature, there is not a default hyperparameter setting for generating the intermediate nearest-neighbor graph for ForceAtlas2. Therefore, we used the hyperparameter settings described in ref. ^29^. A more detailed description is as follows: we constructed a graph of nearest neighbors, where two sample points are connected if and only if one of them is among the five nearest neighbors of the other, defined by Euclidean distance. We fed the graph into ForceAtlas2, initializing the embedding with the first two principal components from PCA, and scaling the initialization to match the scale of ForceAtlas2. We optimized the embedding for 750 iterations, with default settings provided by the implementation^19^. For the version of the implementation of these algorithms being evaluated in this paper, please see Supplementary Note 4.2.

We chose ten published scRNA-transcriptomics datasets to compare the performance of these methods, discussed in Supplementary Note 7. These data are from refs. ^3,6,24–27,30^. The Zheng ERCC and Zheng Monocyte datasets were taken from ref. ^24^. These datasets were derived from lab-made cellular RNAs, and no substantial biological variability was expected. Therefore, they were only used in unsupervised evaluations. The Duo4Eq dataset and Duo8Eq datasets were taken from ref. ^27^. These datasets were derived from purified PBMC subsets, and therefore their labels are reliable as ground truth. For the remaining datasets, the cluster labels were assigned computationally to real-world data and therefore, for these datasets, supervised evaluations may be less reliable, whereas unsupervised evaluations may be more reliable.

Besides the scRNA-seq datasets, we also used two well-studied general datasets to demonstrate the visualization effects of DR algorithms, which are the Mammoth dataset^22,23^, where sample points are 3-dimensional points forming a 3-dimensional mammoth skeleton, and the MNIST handwritten figure dataset^21^, where each sample point is an image belonging to one of ten classes (ten-digit numbers). In addition, we used two synthetic datasets, the Gaussian linear dataset which consists of 20 Gaussians along a line in 50-dimensional space, and the Three-Stage Hierarchical Gaussians dataset (denoted as Hierarchical), which consists of 125 micro clusters that are arranged into 5 meso and 25 macro clusters, for a deeper look at the evaluation of global structure preservation. Details about the generation process are provided in the section “Evaluation 2: global structure preservation” and Supplementary Note 7.1 in the supplementary materials.

All biological datasets used in this paper except the three Zheng datasets and the two Duo datasets were downloaded following links in the original publications. The three Zheng datasets (ERCC, Monocyte and Mouse) were downloaded from 10× genomics website. The Duo4Eq and Duo8Eq datasets were obtained through the bioconductor package DuoClustering2018 in R.

The pre-processed versions of the datasets used in the experiment are either available or can be prepared using the code from our code repository. This repository also contains the code we used to perform the experiments and produce all figures in this manuscript.

For pre-processing the scRNA-seq dataset, we use the packages Seurat^31^ and SCANPY^32^. The pre-processing workflows using these packages are similar, and here we introduce how we do the pre-processing using the Seurat package. The raw count matrix data was usually log-normalized using the “NormalizeData” function in the Seurat package, where the feature counts for each cell are divided by the total counts for that cell, multiplied by a scaling factor, and then log-transformed. The normalization of the scRNA-seq counts is important to correcting for cell-to-cell differences in capture efficiency, sequencing depth, and other technical confounders^33^. Next, a group of, for example, 2000 genes with high variability, were selected as relevant features, using method “FindVariableFeatures” in the Seurat package, where feature variance is calculated on the values standardized using their observed mean and expected variance. Then, the chosen features will be scaled and centered using the “ScaleData” method in Seurat package. Finally, PCA was applied to reduce the dimensionality of the dataset to at most 100 PCs using the “RunPCA” method in the Seurat package.

In the section “Evaluation 4: sensitivity to pre-processing choices”, we studied DR algorithms’ sensitivity to other pre-processing methods, specifically GLM-PCA^28^. GLM-PCA is a pre-processing method that attempts to address the problems caused by the arbitrary choice of pseudocount in the log-normalization, the use of highly variable genes and the PCA step.

### Statistics and reproducibility

We performed statistical analysis using the numpy and scipy packages in Python. For all DR experiments, we use the DR algorithms to process the datasets five times. For evaluation of the DR results, we perform the evaluation on each of the five results, and means and standard errors are provided. All results were analyzed by Student’s unpaired *t* test.

### Reporting summary

Further information on research design is available in the Nature Research Reporting Summary linked to this article.

## Supplementary information

Supplementary Information

Description of Additional Supplementary Files

Supplementary Data 1

Reporting Summary

Featured Image License

Featured Image License

## Data Availability

All datasets used in this paper except the three Zheng datasets and the two Duo datasets were downloaded following links in the original publications. The three Zheng datasets (ERCC, Monocyte, and Mouse) were downloaded from https://support.10xgenomics.com/single-cell-gene-expression/datasets. The Duo4Eq and Duo8Eq datasets were obtained through the bioconductor package DuoClustering2018 in R. The pre-processed version of the datasets used in the experiment is either available or can be prepared using the code from our code repository https://github.com/hyhuang00/scRNA-DR2020. The raw results from the experiment are available in Supplementary Data 1.

## Code availability

The code we used to perform the experiment and produce all the figures in this manuscript is available at https://github.com/hyhuang00/scRNA-DR2020.
