# Supplementary material for: Towards a comprehensive evaluation of dimension reduction methods for transcriptomic data visualization
Source: Commun Biol. 2022 Jul 19;5:719. doi: 10.1038/s42003-022-03628-x (PMC9296444; doi:10.1038/s42003-022-03628-x)
Supplement: Supplementary file 1 — Supplementary Information [file 42003_2022_3628_MOESM1_ESM.pdf]

Supplementary Note 1 Related works.

Dimension reduction algorithms are extremely popular for examining and processing biological datasets[1,2,3,4,5,6], and other types of scientific data such as radio frequency fingerprint data[7], hyperspectral imagery[8], and text documents[9,10].

DR methods can generally be separated into two categories: local and global methods. Global methods include principal components analysis (PCA)[11], multidimensional scaling (MDS)[12], and non-negative matrix factorization (NMF)[13], which aim mainly to preserve distances between pairs of points. In scRNA-seq data analysis, these methods are often used as a preprocessing step (though in this work we focus on data visualization rather than preprocessing).

Comparing to the global methods, local methods are more commonly used for direct visualization of preprocessed biological data. Well-known examples of this category include Isomap[14], Local Linear Embedding (LLE)[15], Laplacian Eigenmap[16], and more recent methods such as t-SNE[17], UMAP[18] and PaCMAP[19]. These methods aim to preserve local neighborhoods (that is, which points are within the k nearest neighbors). Local methods typically preserve cluster structure, but may fail to preserve the overall layout of clusters in the space, and we will give several examples throughout this work of such cases.

The popular algorithm t-SNE[17] interprets distances in high-dimensional space as probabilities of whether the two points of interest should be neighbors. This approach aims to ensure that local neighborhood structure is preserved. Then, during the projection to low dimensions, t-SNE ensures that distances in the low-dimensional space follow a Student t-distribution with a single degree of freedom. Forcing distances in the low-dimensional space to follow such a long-tailed distribution avoids the crowding problem, which is when a large proportion of the distances are almost zero.

Myriad t-SNE variants have aimed to improve t-SNE by enabling faster running times, gaining robustness to hyperparameter changes and improving global structure preservation e.g., ref[20,21], and multiple new algorithms have been created based on the framework of t-SNE. Prominent examples that are used in computational biology include viSNE[22], UMAP[18], and Opt-SNE[23]. With a better initialization created by unsupervised machine learning algorithms (such as spectral clustering) and better loss functions, these algorithms improve global structure preservation and run-time efficiency, though they do not completely solve these problems, as we will show.

Recent studies on DR algorithms shed light on how the loss function affects the rendering of local structure[24], and provide guidance on how to design good loss functions so that the local and global structure can both be preserved simultaneously[19].

Besides general DR methods, two other categories of methods are also used specifically for scRNA-seq data analysis – 1) graph visualization methods and, 2) variational-inference based methods.

Graph visualization methods, such as ForceAtlas2[25] were originally designed to visualize graphs instead of unstructured data. Recently, researchers have been applying these algorithms to k-Nearest Neighbor graphs constructed on the scRNA-seq data, and generating visualizations. Notable variants include SPRING[26] and PHATE[27].

Variational-inference-based methods build statistical models from scRNA-seq data, and assume that gene expression, as measured by unique molecular identifier (UMI) counts, are controlled by a few latent variables. The inferred latent variables are used for downstream analysis. Prominent examples in this category include ZIFA[28], ZINB-WaVE[29], scVI[1], scVIs[2] and GLM-PCA[30]. The focus of our proposed evaluation framework is the visualization performance of these algorithms.

As a result, algorithms that are usually used as pre-processing methods that lead to more than three dimensions, such as global methods and variational-inference-based methods, are not discussed in detail and are not evaluated by the framework in this study. The use of DR methods for preprocessing has been reviewed previously[31].

Supplementary Note 2 Extra figures

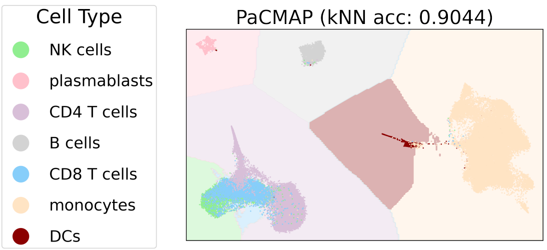

(a) PaCMAP (good local and good global structure)

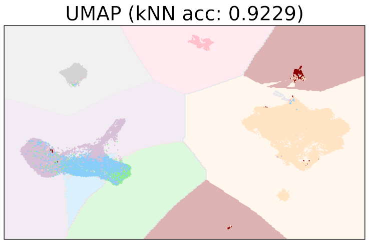

(b) UMAP (good local but bad global structure)

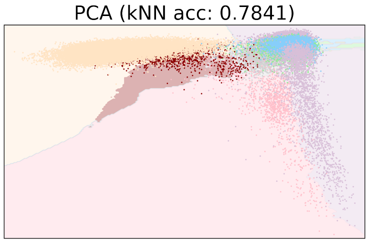

(c) PCA (bad local and good global structure)

Supplementary Figure 1: Illustration of local and global structure preservation and non-preservation, using the Kazer et al.[32] PBMC dataset. The three figures were generated from the same preprocessed data with different DR algorithms (PaCMAP, UMAP or PCA). Boundaries on the figure were generated using k-nearest neighbors: for each background point on the 2D grid, color was assigned based on the majority vote of the k nearest (k=20) data points. The dataset was pre-processed by PCA with 70 PCs generated. Upper left: the PaCMAP visualization preserves both local structure and the global structure. Upper right: UMAP does not preserve global structure well since the different subtypes of DCs are placed separately. (kNN acc is k-nearest neighbors accuracy.) Lower: PCA does not preserve local structure well, reflected by the low kNN (k=5) accuracy. Thus for PCA, points from different classes are mixed together, resulting in unclear boundaries.

Supplementary Figure 1 shows DR results on the Kazer et al.[32] PBMC dataset with PaCMAP, UMAP and PCA, and demonstrates different type of structures we discuss in Section 2.1 and Section 2.2. Supplementary Figure 2 shows the DR results on the Kang et al.[33] dataset. Supplementary Figure 3 shows DR results on the MNIST dataset, with the supervised kNN evaluation. Supplementary Figure 4 shows DR results of art-SNE on the Kazer et al.[32] HIV-infection PBMC dataset, Stuart et al.[34] bone marrow dataset, and MNIST dataset.

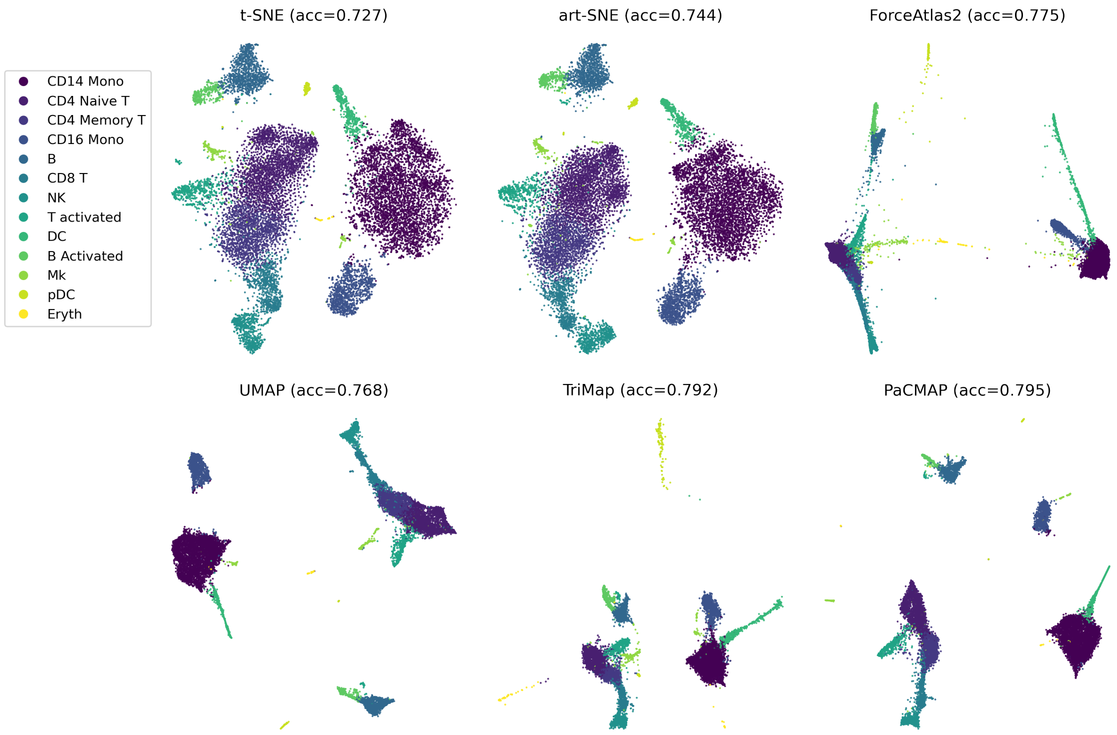

Supplementary Figure 2: Measuring global structure preservation with random triplet accuracy. Visualization of the Kang et al.[33] dataset. The triplet accuracy score (acc) is the random triplet accuracy mentioned in Section 2.2. TriMap and PaCMAP achieve the highest level of global structure out of all the DR algorithms.

Supplementary Note 3 Robustness of DR algorithm with different runs

DR algorithms can behave differently under different random seeds based on the implementations. To ensure the results we obtained are generally useful, we have perform our experiments on the embeddings generated with five different random seeds for each algorithm. Nevertheless, it is still necessary to provide a qualitative analysis on the robustness of each algorithm. Here in Supplementary Figure 5, we choose to use the Kazer et al.[32] dataset that is used for Figure 5 to perform a qualitative analysis on the effect of different random seeds on t-SNE, UMAP, TriMap and PaCMAP. From the results we can see that t-SNE, TriMap and PaCMAP are relatively consistent under the selected implementations, whereas UMAP sometimes generates different embeddings. In addition, UMAP fails to preserve the local structure of some clusters in some rare cases.

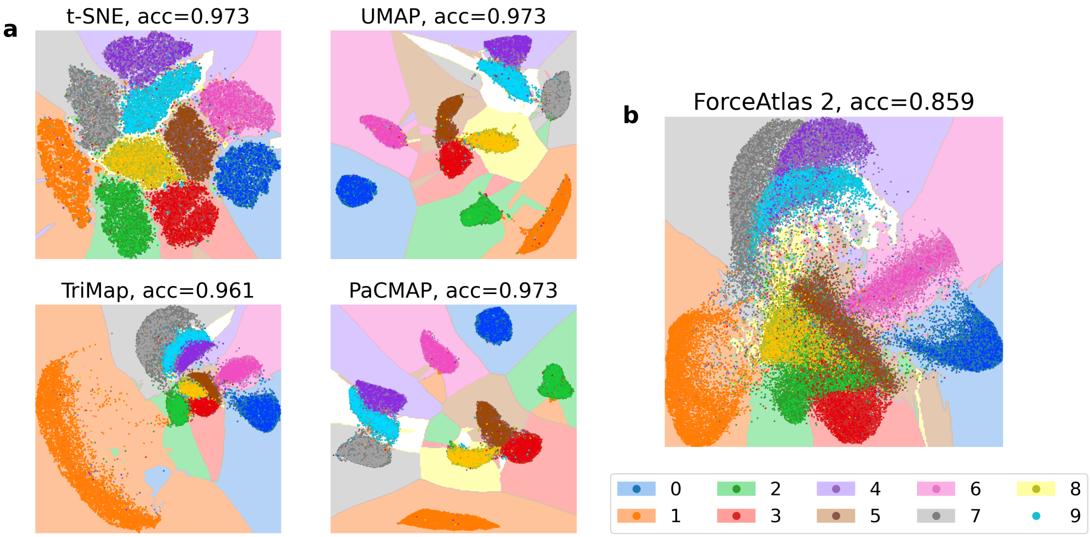

Supplementary Figure 3: Measuring local structure preservation with kNN (k=5) supervised classification. Visualizations of MNIST dataset generated by different algorithms. The accuracy score (acc) and decision boundaries were created by a kNN ($k = 5$) classifier, where 90% of the dataset was used for the purpose of computing and visualizing decision boundaries (lighter color shades). The remaining 10% of the data were points on which test accuracies were computed, using the 90% of the data as possible neighbors (darker color shades). We conducted the experiment this way to be consistent with the training/test split on the SVM experiment. Similar to the SVM experiment, here ForceAtlas 2 does not preserve local structure well.

Supplementary Note 4 Notes on Implementation

Supplementary Note 4.1 Difference between t-SNE variants

The default openTSNE settings incorporate advice from art-SNE[35], so except for perplexity, the other hyperparameter settings are similar. The default openTSNE adopts a perplexity of 30, whereas for art-SNE, the perplexity is a combination of 30 and n/100 where n is the sample size. For t-SNE, we used the python-centric openTSNE implementation, whereas for art-SNE, we used the FIt-SNE implementation that is more widely used in the biological domain. The reason for such choices is because both implementations are widely used and they could have different behavior, so we include both of them for experiments.

Supplementary Note 4.2 Version of software packages being evaluated

The packages used to preprocess the scRNA-seq data are Seurat and SCANPY. The packages used to implement DR algorithms are open-TSNE v0.3.13 for t-SNE, FItSNE v1.2.1 for t-SNE with the hyperparameters described in[35](art-SNE), umap-learn 0.5.0 for UMAP, fa2 v0.3.5 for ForceAtlas2,

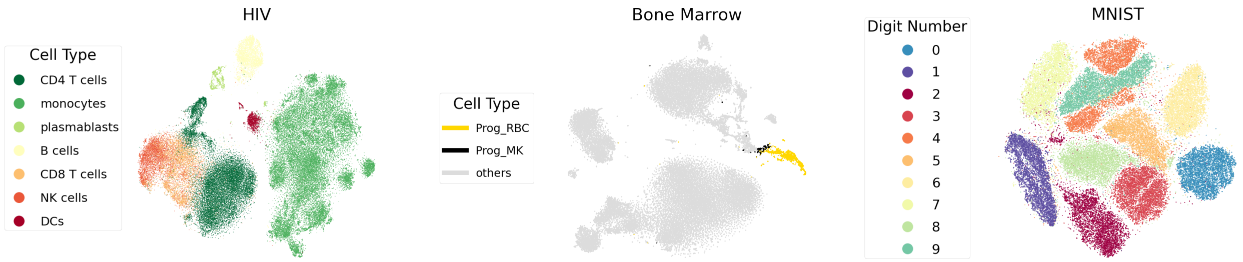

Supplementary Figure 4: art-SNE's DR results using the Kazer et al.[32] HIV-infection PBMC dataset, Stuart et al.[34] bone marrow dataset, and hand-written digit MNIST dataset.

PaCMAP 0.3.0 for PaCMAP, TriMap 0.1.0 for TriMap, and PHATE 1.0.7 for PHATE.

Supplementary Note 5 Local structure preservation evaluation procedure and results

Supplementary Note 5.1 Local Supervised evaluation

Supplementary Table 1: Measuring local structure preservation with SVM supervised classification (for datasets that possess class labels). SVM accuracy and standard deviation over 10 folds are reported in the table. Here methods that focus on local structure, such as PaCMAP, t-SNE and art-SNE, perform well. **Bold** is used for the best performance, *italics* is used when the result is not significantly different from the best one (defined by a one-sided t-test with P value of 0.05). Mean and standard deviation are computed over 5 repetitions.

Dataset	PCA	t-SNE	art-SNE	ForceAtlas2	UMAP	TriMap	PaCMAP	PHATE
Duo 4Eq	0.587 ± 0.000	*0.758 ± 0.003*	**0.760 ± 0.002**	0.676 ± 0.004	0.728 ± 0.008	0.729 ± 0.002	*0.757 ± 0.009*	0.657 ± 0.015
Duo 8Eq	0.610 ± 0.000	0.789 ± 0.003	0.784 ± 0.002	0.740 ± 0.001	0.784 ± 0.004	0.748 ± 0.009	**0.807 ± 0.002**	0.743 ± 0.005
Kazer	0.817 ± 0.000	0.936 ± 0.001	0.935 ± 0.000	0.918 ± 0.000	0.935 ± 0.003	0.938 ± 0.000	**0.940 ± 0.002**	0.921 ± 0.004
Muraro	0.663 ± 0.000	0.953 ± 0.001	0.953 ± 0.001	0.918 ± 0.000	0.946 ± 0.001	0.952 ± 0.001	**0.958 ± 0.000**	0.953 ± 0.003
Kang	0.733 ± 0.000	0.955 ± 0.002	0.952 ± 0.002	0.945 ± 0.000	0.953 ± 0.001	0.954 ± 0.001	**0.957 ± 0.001**	0.916 ± 0.004
Stuart	0.460 ± 0.000	0.841 ± 0.002	0.837 ± 0.002	0.817 ± 0.001	0.837 ± 0.004	0.832 ± 0.002	**0.848 ± 0.001**	0.764 ± 0.012
Zheng Mouse	0.379 ± 0.001	**0.840 ± 0.001**	–	0.716 ± 0.000	0.802 ± 0.011	0.728 ± 0.000	0.791 ± 0.008	–
Cao	0.286 ± 0.000	**0.742 ± 0.006**	–	0.593 ± 0.000	*0.741 ± 0.012*	0.639 ± 0.003	0.720 ± 0.013	–
Mammoth	0.863 ± 0.000	**0.943 ± 0.001**	0.933 ± 0.000	0.867 ± 0.000	0.925 ± 0.012	0.894 ± 0.004	0.901 ± 0.016	0.861 ± 0.006
MNIST	0.470 ± 0.000	0.971 ± 0.000	0.950 ± 0.000	0.827 ± 0.000	0.972 ± 0.000	0.963 ± 0.001	**0.975 ± 0.000**	0.859 ± 0.017
Hierarchical	0.738 ± 0.000	**1.000 ± 0.000**	**1.000 ± 0.000**	**1.000 ± 0.000**	**1.000 ± 0.000**	**1.000 ± 0.000**	**1.000 ± 0.000**	0.963 ± 0.019

Supplementary Note 5.2 Local Unsupervised evaluation

The evaluation procedure for local unsupervised evaluation is as follows:

- Step 1) Find k nearest neighbors for each point in the preprocessed dataset. For each point with index i, we have a set of neighbors $\mathbf{N}(i)$, which is of size k.
- Step 2) Perform dimension reduction on the $\mathbf{x}_i$'s. Here, we hope that the DR method keeps the neighbor relationships from the high dimensional space.

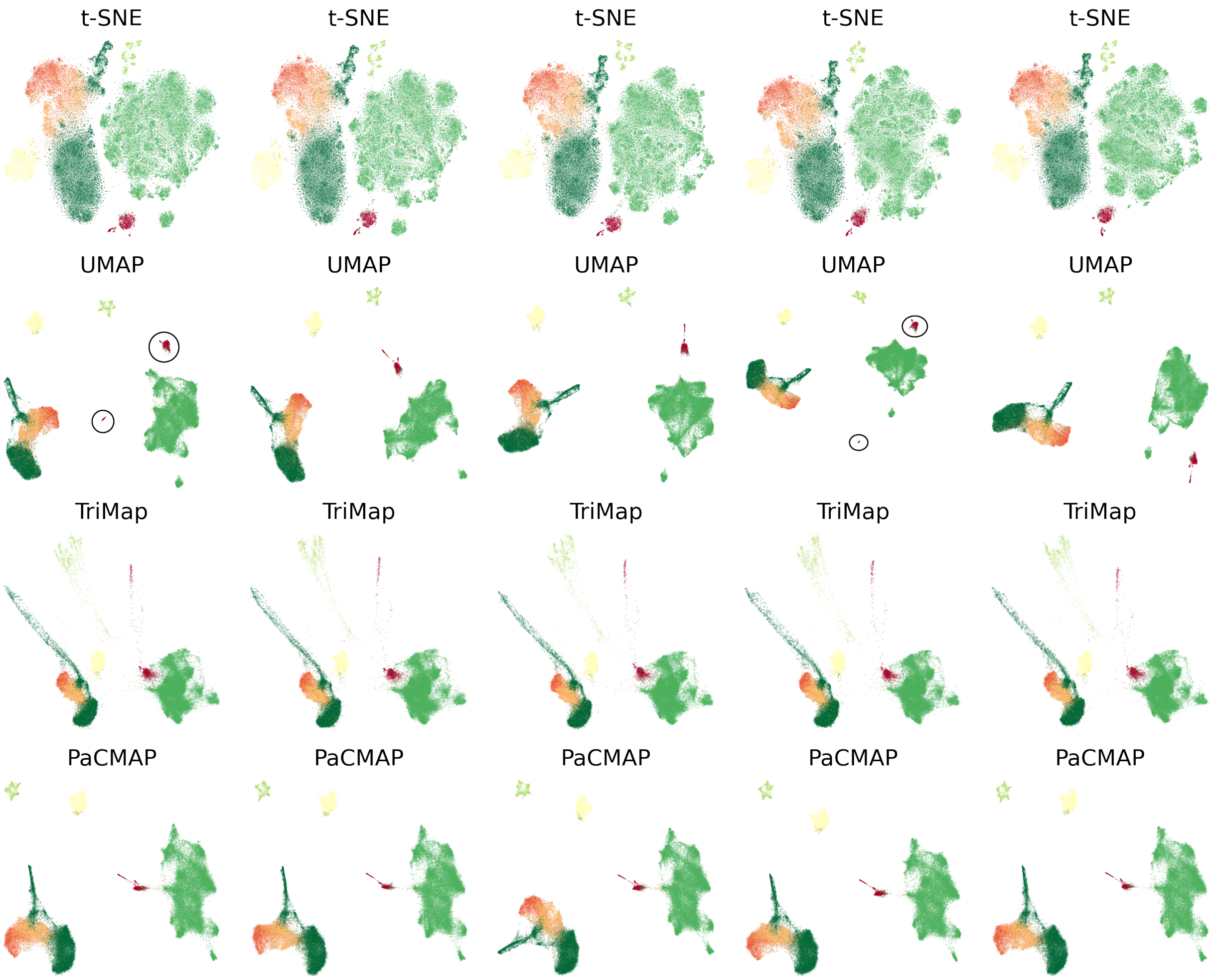

Supplementary Figure 5: DR algorithms' performances for different runs. All the results were generated with default parameters for consistency. We can see that UMAP is not very robust, and in some cases it has generated incorrect local structure (see circled red clusters on the first and the fourth column.)

- Step 3) Find k nearest neighbors for each point in the low-dimensional space. For each point with index i, we have a new set of neighbors $\mathbf{N}'(i)$.
- Step 4) Compute the proportion of intersection between the two sets $\mathbf{N}(i)$ and $\mathbf{N}'(i)$ for each index i, and then compute the average proportion of neighbors preserved.

Supplementary Table 3 summarizes the preservation of neighborhood for different visualizations of all datasets.

Supplementary Note 6 Running time table

Supplementary Table 4 contains the running time for each algorithm.

Supplementary Table 2: kNN Accuracy for datasets that possess class labels, for k=5. kNN accuracy and standard deviation over 10 folds are reported in the table. Similar to the SVM experiment, local methods such as t-SNE, art-SNE and PaCMAP perform well. Mean and standard deviation are computed over 5 repetitions. **Bold** is used for the best performance, *italics* is used when the result is not significantly different from the best one (defined by a one-sided t-test with P value of 0.05).

Dataset	PCA	t-SNE	art-SNE	ForceAtlas2	UMAP	TriMap	PaCMAP	PHATE
Duo 4Eq	0.516 ± 0.000	*0.747 ± 0.003*	**0.748 ± 0.002**	0.690 ± 0.002	0.722 ± 0.008	0.713 ± 0.006	0.740 ± 0.004	0.661 ± 0.007
Duo 8Eq	0.568 ± 0.000	*0.798 ± 0.003*	**0.801 ± 0.005**	0.740 ± 0.003	0.772 ± 0.007	0.762 ± 0.004	0.788 ± 0.006	0.713 ± 0.007
Kazer	0.791 ± 0.000	0.932 ± 0.000	0.931 ± 0.001	0.922 ± 0.000	0.933 ± 0.000	0.933 ± 0.001	**0.935 ± 0.001**	0.918 ± 0.005
Muraro	0.662 ± 0.000	0.961 ± 0.001	*0.961 ± 0.001*	**0.962 ± 0.001**	0.957 ± 0.001	0.955 ± 0.002	*0.961 ± 0.002*	0.954 ± 0.002
Kang	0.711 ± 0.000	0.959 ± 0.001	0.959 ± 0.001	0.953 ± 0.000	0.957 ± 0.001	0.959 ± 0.001	**0.962 ± 0.001**	0.925 ± 0.005
Stuart	0.420 ± 0.000	*0.845 ± 0.001*	0.845 ± 0.001	0.830 ± 0.001	0.837 ± 0.001	0.838 ± 0.001	**0.846 ± 0.001**	0.791 ± 0.003
Zheng Mouse	0.317 ± 0.000	**0.901 ± 0.006**	–	0.709 ± 0.004	0.818 ± 0.005	0.734 ± 0.011	0.774 ± 0.007	–
Cao	0.210 ± 0.000	**0.839 ± 0.008**	–	0.579 ± 0.008	0.773 ± 0.005	0.668 ± 0.002	0.743 ± 0.009	–
Mammoth	0.883 ± 0.000	**0.984 ± 0.000**	**0.984 ± 0.000**	0.961 ± 0.000	**0.984 ± 0.001**	0.966 ± 0.001	0.968 ± 0.004	0.960 ± 0.002
MNIST	0.414 ± 0.000	**0.976 ± 0.000**	0.972 ± 0.000	0.821 ± 0.001	0.973 ± 0.000	0.964 ± 0.001	**0.976 ± 0.000**	0.873 ± 0.015
Hierarchical	0.719 ± 0.000	**1.000 ± 0.000**	**1.000 ± 0.000**	**1.000 ± 0.000**	**1.000 ± 0.000**	**1.000 ± 0.000**	**1.000 ± 0.000**	0.988 ± 0.007

Supplementary Table 3: Unsupervised local evaluation; fraction of neighborhood (k=30 neighbors) that are successfully preserved. Mean and standard deviation are computed over 5 repetitions. **Bold** is used for the best performance, *italics* is used when the result is not significantly different from the best one (defined by a one-sided t-test with P value of 0.05). Here, t-SNE and art-SNE preserve the most neighborhood information from the high dimensional space.

Dataset	PCA	t-SNE	art-SNE	ForceAtlas2	UMAP	TriMap	PaCMAP	PHATE
Zheng ERCC	0.090 ± 0.000	**0.197 ± 0.001**	0.182 ± 0.002	0.091 ± 0.001	0.175 ± 0.002	0.181 ± 0.002	0.142 ± 0.001	0.102 ± 0.001
Zheng Monocyte	0.077 ± 0.000	**0.146 ± 0.001**	*0.145 ± 0.000*	0.086 ± 0.000	0.108 ± 0.001	0.116 ± 0.001	0.100 ± 0.001	0.081 ± 0.000
Duo 4Eq	0.089 ± 0.000	**0.211 ± 0.001**	*0.210 ± 0.001*	0.138 ± 0.000	0.163 ± 0.001	0.161 ± 0.001	0.150 ± 0.002	0.123 ± 0.002
Duo 8Eq	0.089 ± 0.000	**0.211 ± 0.001**	0.209 ± 0.000	0.138 ± 0.000	0.163 ± 0.001	0.161 ± 0.000	0.148 ± 0.001	0.123 ± 0.001
Kazer	0.011 ± 0.000	**0.171 ± 0.000**	0.149 ± 0.000	0.051 ± 0.000	0.072 ± 0.000	0.062 ± 0.000	0.061 ± 0.001	0.047 ± 0.001
Muraro	0.118 ± 0.000	**0.518 ± 0.002**	0.505 ± 0.001	0.381 ± 0.001	0.469 ± 0.002	0.446 ± 0.005	0.424 ± 0.004	0.323 ± 0.002
Kang	0.034 ± 0.000	**0.217 ± 0.000**	0.211 ± 0.000	0.118 ± 0.000	0.135 ± 0.001	0.134 ± 0.001	0.125 ± 0.001	0.104 ± 0.001
Stuart	0.022 ± 0.000	**0.176 ± 0.000**	0.162 ± 0.000	0.083 ± 0.000	0.103 ± 0.001	0.095 ± 0.001	0.095 ± 0.001	0.082 ± 0.001
Zheng Mouse	0.002 ± 0.000	**0.101 ± 0.001**	–	0.021 ± 0.001	0.033 ± 0.001	0.024 ± 0.001	0.025 ± 0.000	–
Cao	0.001 ± 0.000	**0.073 ± 0.001**	–	0.011 ± 0.000	0.024 ± 0.001	0.014 ± 0.001	0.019 ± 0.001	–
Mammoth	0.462 ± 0.000	0.773 ± 0.001	**0.787 ± 0.001**	0.547 ± 0.000	0.709 ± 0.002	0.596 ± 0.004	0.640 ± 0.007	0.434 ± 0.004
MNIST	0.017 ± 0.000	**0.345 ± 0.000**	0.307 ± 0.000	0.081 ± 0.000	0.156 ± 0.001	0.123 ± 0.000	0.135 ± 0.000	0.109 ± 0.002
Hierarchical	0.061 ± 0.000	**0.148 ± 0.000**	0.126 ± 0.000	0.108 ± 0.000	0.078 ± 0.000	0.086 ± 0.000	0.076 ± 0.000	0.077 ± 0.002

Supplementary Note 7 Details on Datasets

We picked ten published scRNA-seq transcriptomics datasets, shown in Supplementary Table 5. Supplementary Figure 6 provides a visualization of all datasets generated by PaCMAP.

Besides the scRNA-seq datasets, we also used two well-studied general datasets to demonstrate the visualization effects of DR algorithms, which are the Mammoth dataset[39,40] and the MNIST handwritten figure dataset[41]. In addition, we used two synthetic datasets, the Gaussian linear dataset and the Three Stage Hierarchical Gaussians dataset (denoted as Hierarchical) for a deeper look at the evaluation of global structure preservation. Details about the generation process are provided in Section 2.2 and Supplementary Note 7.1 in the supplementary materials.

For preprocessing the scRNA-seq dataset, we use the packages Seurat[42] and SCANPY[43]. The preprocessing workflows using these packages are similar, and here we introduce how we do the preprocessing using the Seurat package. The raw count matrix data was usually log-normalized using the "NormalizeData" function in the Seurat package, where the feature counts for each cell are divided by the total counts for that cell, multiplied by a scaling factor and then log-transformed. The

Supplementary Table 4: Running time comparison. Mean and standard deviation are computed over 5 repetitions. **Bold** is used for the best performance, *italics* is used when the result is not significantly different from the best one (defined by a one-sided t-test with P value of 0.05). For each algorithm, the running time is defined as the time required to transform the preprocessed dataset with shape $N \times d$ into a low-dimensional embedding with shape $N \times 2$, using the default convergence criteria for each algorithm. Therefore, for ForceAtlas2, the nearest-neighbor graph construction time is also included for fairness. For the Zheng Mouse and Cao datasets, art-SNE ran out of memory, and PHATE cannot finish these datasets within a time limit of 24 hours.

Dataset	size	t-SNE	art-SNE	ForceAtlas2	UMAP	TriMap	PaCMAP	Phate
Zheng ERCC	1015	00:00:11	00:00:08	**00:00:01**	00:00:05	**00:00:01**	**00:00:01**	00:00:02
Zheng Monocyte	2612	00:00:15	00:00:10	00:00:04	00:00:10	00:00:03	**00:00:02**	00:00:32
Duo 4Eq	3994	00:00:17	00:00:12	00:00:06	00:00:17	00:00:04	**00:00:03**	00:00:39
Duo 8Eq	3994	00:00:16	00:00:12	00:00:06	00:00:17	00:00:04	**00:00:03**	00:00:39
Kazer	59286	00:02:14	00:19:15	00:02:19	00:01:01	00:01:04	**00:00:38**	00:08:56
Muraro	2282	00:00:17	00:00:14	00:00:03	00:00:09	00:00:03	**00:00:02**	00:00:31
Kang	13999	00:00:43	00:01:03	00:00:26	00:00:17	00:00:13	**00:00:08**	00:00:55
Stuart	30672	00:01:26	00:05:35	00:01:12	00:00:34	00:00:32	**00:00:20**	00:01:51
Zheng Mouse	1306127	01:13:41	–	01:42:08	01:00:02	00:30:38	**00:20:52**	–
Cao	2058652	02:43:38	–	03:04:53	**00:34:25**	01:08:16	00:42:20	–
Mammoth	10000	00:00:58	00:00:45	00:00:15	00:00:20	00:00:09	**00:00:06**	00:00:45
MNIST	70000	00:03:05	00:33:37	00:02:29	00:01:05	00:01:16	**00:00:49**	00:13:11
Hierarchical	62500	00:01:54	00:14:07	00:01:37	00:00:56	00:01:04	**00:00:36**	00:01:34

Supplementary Table 5: scRNA-seq datasets we used in this paper. Cells are from human tissue for the datasets of Kazer et al.[32], Kang et al.[33], Stuart et al.[34], Duò et al.[36], Muraro et al.[37].

Abbreviation	Author	Tissue	Cells	Genes	Notes
Zheng ERCC	[38]	–	N/A	N/A	Spike-in only; 1015 entries with 91 columns
Zheng Monocyte	[38]	Monocytes	2612	14175	1 cell type; biological negative control
Duo 4Eq	[38] [36]	PBMCs	3994	15568	4 equal clusters of fluorescence activated cell sorting
Duo 8Eq	[38] [36]	PBMCs	3994	15716	8 equal clusters of fluorescence activated cell sorting
Kazer	[32]	PBMCs	59286	16980	Authors computationally identified 7 types
Muraro	[37]	Pancreas	2282	18962	Authors computationally identified 9 types
Kang	[33]	PBMCs	13999	14053	Authors computationally identified 13 types
Stuart	[34]	Bone Marrow	30672	17009	Authors computationally identified 25 types
Zheng Mouse	[38]	Mouse brain	1306127	27998	Benchmarking Computational speed
Cao	[3]	Mouse Embryo	2058652	26183	Benchmarking computational speed

normalization of the scRNA-seq counts is important to correcting for cell-to-cell differences in capture efficiency, sequencing depth, and other technical confounders[44]. Next, a group of, for example, 2000 genes with high variability, were selected as relevant features, using method "FindVariableFeatures" in the Seurat package, where feature variance is calculated on the values standardized using their observed mean and expected variance. Then, the chosen features will be scaled and centered using the "ScaleData" method in Seurat package. Finally PCA was applied to reduce the dimensionality of the dataset to at most 100 PCs using the "RunPCA" method in the Seurat package.

In Section 2.4, we study DR algorithms' sensitivity to other preprocessing methods, specifically GLM-PCA[30]. GLM-PCA is a preprocessing method that attempts to address the problems caused by the arbitrary choice of pseudocount in the log-normalization, the use of highly variable genes and the PCA step.

We added two simulated datasets that allow us to determine whether DR methods preserve known structure. These are the Gaussian Linear Dataset and the Three-Stage Hierarchical Dataset.

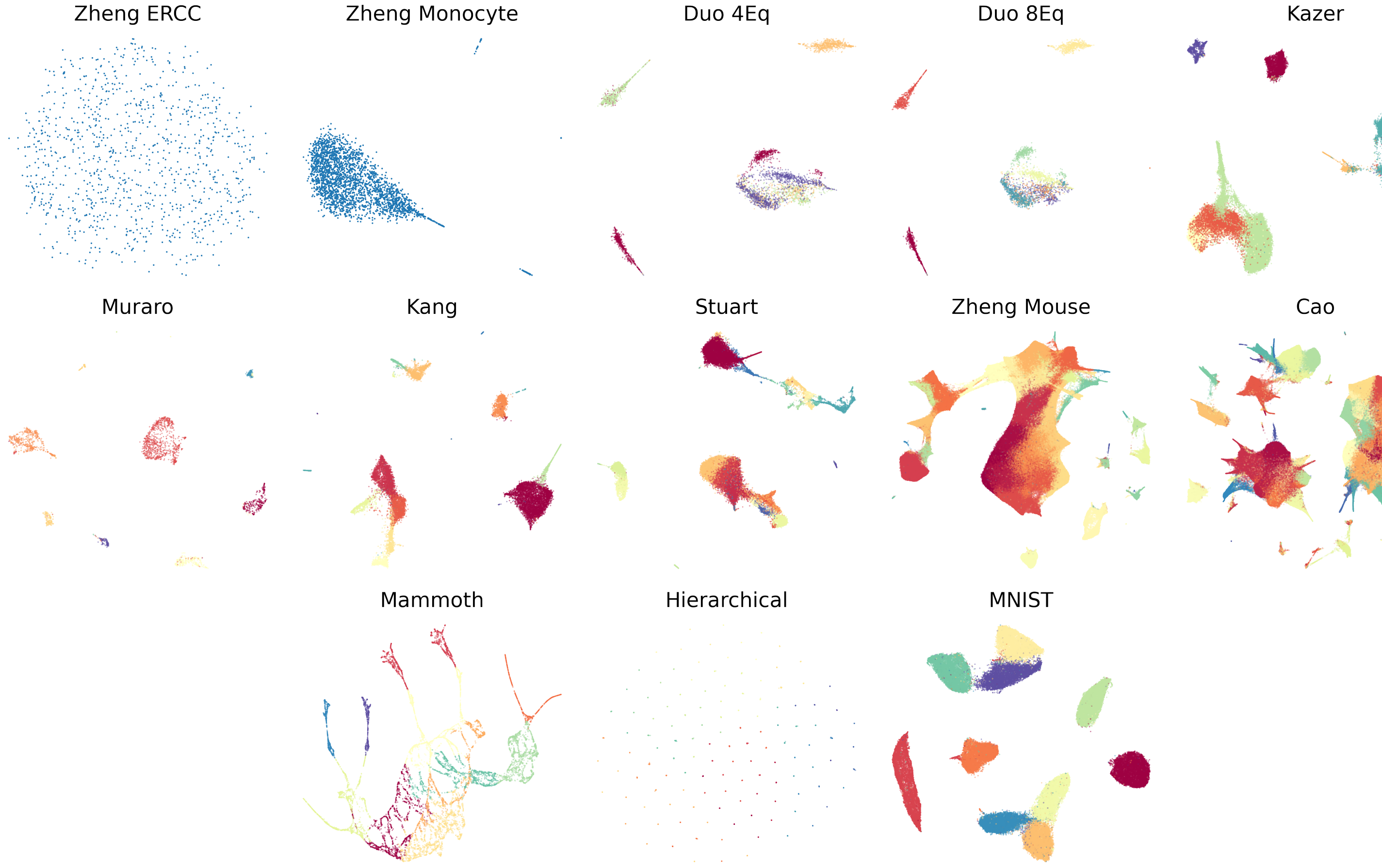

Supplementary Figure 6: Visualization of all datasets involved in this paper. All the results are generated with PaCMAP, and colored with the original labels.

Gaussian Linear Dataset: Subplot (c) in Figure 4 shows DR results for the Gaussian Linear dataset. The Gaussian Linear dataset was created to demonstrate different DR algorithms' global structure preservation capacity. The synthetic dataset is generated from 20 isotropic Gaussians existing in 50-dimensional space, separated by equal distances along a line. Although most DR algorithms successfully capture the general left-to-right arrangement of the clusters, popular DR methods such as t-SNE cannot capture this global information under default parameter settings.

Three-Stage Hierarchical Gaussians Dataset: Another hierarchical Gaussian dataset was created to simulate the differentiation of cells, similar to previous reports[19,35]. This dataset consists of 125 micro clusters, arranged into 5 macro and 25 meso clusters. Each micro cluster includes 500 observations. See Supplementary Note 7.1 for the detailed data generation process. Subplot (d) in Figure 4 summarizes the visualization results of different DR algorithms for this dataset. We can see that t-SNE and UMAP fail to preserve the global structure since micro clusters from the same macro clusters are not placed together, whereas PaCMAP and TriMAP successfully retain this information. ForceAtlas2 performs very well here.

Supplementary Note 7.1 Generation process for the Three-layer Hierarchical Gaussians dataset

The Three-layer Hierarchical Gaussians dataset was first introduced by Wang et al.[19] to check the ability of different DR algorithms to preserve multi-scale, complicated hierarchical structures. The data generation process was as follows:

1. We sampled five macro cluster centers from a 50-dimensional multivariate normal distribution with zero mean ($\mathbf{0}_{50}$) and a covariance matrix that is equal to the identity matrix multiplied by 10000 (i.e., $10000 \times \mathbf{1}_{50}$).
2. We then sampled five meso cluster centers for each of the macro clusters (5×5 =25 in total) using a 50-dimensional multivariate normal distribution whose mean values are the macro cluster centers and whose covariance matrices are equal to the identity matrix multiplied by 1000 (i.e., $1000 \times \mathbf{1}_{50}$).
3. We then sampled five micro cluster centers for each of the meso clusters (25×5 =125 in total) using a 50-dimensional multivariate normal distribution whose mean values are the meso cluster centers and whose covariance matrices are equal to the identity matrix multiplied by 100 (i.e., $100 \times \mathbf{1}_{50}$).
4. Finally, for each of the one hundred and twenty five micro cluster centers, we sampled five hundred observations from a 50-dimensional multivariate normal distribution whose mean values are the micro cluster centers and whose covariance matrices are equal to $10 \times \mathbf{1}_{50}$.

Supplementary Note 8 Global structure preservation results

We utilized the random triplet accuracy, distance Spearman correlation, k-nearest classes preservation, and centroid distance correlation to quantitatively evaluate the global structure preservation for DR methods. Supplementary Table 6, Supplementary Table 7, Supplementary Table 8, Supplementary Table 9 summarize the result for different visualizations of all datasets.

Supplementary Table 6: Random Triplet Accuracy calculated over different visualizations of multiple datasets. Mean and standard deviation are computed over 5 repetitions. **Bold** is used for the best performance, *italics* is used when the result is not significantly different from the best one (defined by a one-sided t-test with P value of 0.05). Traditional methods like PCA perform well.

DATASET	PCA	T-SNE	ART-SNE	FORCEATLAS2	UMAP	TRIMAP	PACMAP	PHATE
ZHENG ERCC	0.605 ± 0.006	0.603 ± 0.006	0.582 ± 0.006	0.576 ± 0.003	0.576 ± 0.008	**0.619 ± 0.008**	0.598 ± 0.007	0.592 ± 0.008
ZHENG MONOCYTE	**0.741 ± 0.003**	0.688 ± 0.004	0.686 ± 0.002	0.728 ± 0.003	0.690 ± 0.005	0.725 ± 0.004	0.725 ± 0.004	0.723 ± 0.002
DUO 4EQ	**0.804 ± 0.001**	0.693 ± 0.003	0.697 ± 0.002	0.768 ± 0.001	0.691 ± 0.021	0.779 ± 0.002	0.767 ± 0.013	0.724 ± 0.009
DUO 8EQ	**0.808 ± 0.003**	0.696 ± 0.002	0.697 ± 0.003	0.768 ± 0.002	0.718 ± 0.017	0.784 ± 0.005	0.779 ± 0.003	0.729 ± 0.009
KAZER	0.820 ± 0.001	0.735 ± 0.010	0.762 ± 0.003	0.803 ± 0.001	0.750 ± 0.012	**0.822 ± 0.000**	0.791 ± 0.001	0.816 ± 0.002
MURARO	0.759 ± 0.004	0.694 ± 0.004	0.729 ± 0.005	**0.777 ± 0.001**	0.699 ± 0.009	0.757 ± 0.006	0.744 ± 0.006	0.753 ± 0.004
KANG	**0.812 ± 0.001**	0.727 ± 0.003	0.744 ± 0.004	0.777 ± 0.001	0.768 ± 0.012	0.792 ± 0.003	0.795 ± 0.003	0.794 ± 0.003
STUART	0.728 ± 0.001	0.711 ± 0.003	0.671 ± 0.005	0.713 ± 0.001	0.662 ± 0.009	**0.769 ± 0.001**	0.754 ± 0.001	0.727 ± 0.006
ZHENG MOUSE	0.719 ± 0.000	0.697 ± 0.000	–	0.719 ± 0.000	0.722 ± 0.003	**0.768 ± 0.000**	0.762 ± 0.003	–
CAO	0.704 ± 0.000	0.663 ± 0.001	–	0.702 ± 0.000	0.626 ± 0.012	**0.756 ± 0.000**	0.679 ± 0.003	–
MAMMOTH	**0.962 ± 0.001**	0.819 ± 0.001	0.830 ± 0.002	0.947 ± 0.001	0.807 ± 0.004	0.862 ± 0.001	0.869 ± 0.004	0.655 ± 0.006
MNIST	**0.685 ± 0.001**	0.623 ± 0.001	0.643 ± 0.001	0.635 ± 0.000	0.609 ± 0.003	0.603 ± 0.000	0.614 ± 0.006	0.619 ± 0.002
HIERARCHICAL	**0.868 ± 0.001**	0.693 ± 0.001	0.742 ± 0.003	0.842 ± 0.001	0.504 ± 0.008	0.648 ± 0.001	0.805 ± 0.002	0.506 ± 0.002

Supplementary Table 7: Distance Spearman Correlation: spearman correlation between pairwise distances in high- and low-dimensional embeddings. Mean and standard deviation are computed over 5 repetitions. **Bold** is used for the best performance, *italics* is used when the result is not significantly different from the best one (defined by a one-sided t-test with P value of 0.05).

Dataset	PCA	t-SNE	art-SNE	ForceAtlas2	UMAP	TriMap	PaCMAP	PHATE
Zheng ERCC	0.304 ± 0.000	0.308 ± 0.004	0.248 ± 0.006	0.221 ± 0.009	0.214 ± 0.006	**0.435 ± 0.008**	0.292 ± 0.017	0.272 ± 0.001
Zheng Monocyte	0.763 ± 0.000	0.599 ± 0.003	0.570 ± 0.005	0.758 ± 0.000	0.670 ± 0.003	**0.770 ± 0.003**	0.706 ± 0.003	0.754 ± 0.001
Duo 4Eq	**0.837 ± 0.000**	0.576 ± 0.002	0.588 ± 0.005	0.709 ± 0.000	0.550 ± 0.070	0.732 ± 0.004	0.717 ± 0.012	0.617 ± 0.008
Duo 8Eq	**0.837 ± 0.000**	0.575 ± 0.003	0.586 ± 0.003	0.709 ± 0.000	0.616 ± 0.078	0.734 ± 0.004	0.728 ± 0.003	0.622 ± 0.008
Kazer	0.828 ± 0.000	0.631 ± 0.034	0.697 ± 0.007	0.786 ± 0.000	0.673 ± 0.033	**0.843 ± 0.001**	0.772 ± 0.004	0.793 ± 0.004
Muraro	**0.867 ± 0.000**	0.460 ± 0.008	0.551 ± 0.004	0.860 ± 0.000	0.519 ± 0.036	0.741 ± 0.005	0.602 ± 0.011	0.788 ± 0.002
Kang	**0.796 ± 0.000**	0.581 ± 0.008	0.616 ± 0.008	0.700 ± 0.000	0.670 ± 0.031	0.765 ± 0.003	0.746 ± 0.003	0.736 ± 0.005
Stuart	0.695 ± 0.000	0.551 ± 0.006	0.422 ± 0.016	0.679 ± 0.000	0.433 ± 0.011	**0.844 ± 0.001**	0.648 ± 0.003	0.607 ± 0.009
Zheng Mouse	0.742 ± 0.000	0.522 ± 0.001	–	0.685 ± 0.000	0.714 ± 0.008	**0.871 ± 0.001**	0.714 ± 0.000	–
Cao	0.583 ± 0.000	0.479 ± 0.002	–	0.617 ± 0.000	0.442 ± 0.054	**0.798 ± 0.001**	0.523 ± 0.003	–
Mammoth	**0.992 ± 0.000**	0.761 ± 0.001	0.791 ± 0.001	0.986 ± 0.000	0.814 ± 0.009	0.839 ± 0.003	0.877 ± 0.008	0.328 ± 0.022
MNIST	**0.559 ± 0.000**	0.366 ± 0.001	0.432 ± 0.001	0.381 ± 0.000	0.328 ± 0.008	0.198 ± 0.001	0.323 ± 0.022	0.341 ± 0.012
Hierarchical	**0.891 ± 0.000**	0.524 ± 0.004	0.639 ± 0.006	0.854 ± 0.000	0.015 ± 0.023	0.419 ± 0.004	0.806 ± 0.005	0.025 ± 0.006

Supplementary Table 8: k-nearest classes preservation. Mean and standard deviation are computed over 5 repetitions. **Bold** is used for the best performance, *italics* is used when the result is not significantly different from the best one (defined by a one-sided t-test with P value of 0.05). We choose k to be a dynamic value that depends on the $k = \lfloor \frac{C+2}{4} \rfloor$, where C is the number of classes in the dataset. PCA and ForceAtlas2 have better performance across all datasets.

Dataset	PCA	t-SNE	art-SNE	ForceAtlas2	UMAP	TriMap	PaCMAP	PHATE
Duo 4Eq	**1.000 ± 0.000**	0.750 ± 0.000	0.500 ± 0.000	**1.000 ± 0.000**	0.400 ± 0.200	0.800 ± 0.100	0.450 ± 0.100	0.500 ± 0.000
Duo 8Eq	0.812 ± 0.000	0.750 ± 0.000	0.688 ± 0.000	**0.938 ± 0.000**	0.800 ± 0.100	0.738 ± 0.025	0.787 ± 0.031	0.812 ± 0.040
Kazer	**0.857 ± 0.000**	0.686 ± 0.035	0.700 ± 0.029	0.786 ± 0.000	0.686 ± 0.035	0.786 ± 0.000	0.714 ± 0.000	0.757 ± 0.035
Muraro	0.500 ± 0.000	0.333 ± 0.000	0.500 ± 0.000	0.500 ± 0.000	0.511 ± 0.042	**0.578 ± 0.027**	0.467 ± 0.075	*0.567 ± 0.022*
Kang	0.590 ± 0.000	0.446 ± 0.021	0.487 ± 0.016	**0.615 ± 0.000**	0.513 ± 0.036	*0.600 ± 0.031*	0.533 ± 0.019	0.574 ± 0.013
Stuart	0.540 ± 0.000	0.596 ± 0.003	0.548 ± 0.008	0.551 ± 0.002	0.577 ± 0.007	0.595 ± 0.011	**0.652 ± 0.008**	0.606 ± 0.003
Zheng Mouse	0.469 ± 0.000	0.433 ± 0.004	–	0.443 ± 0.002	0.473 ± 0.008	0.477 ± 0.002	**0.538 ± 0.003**	–
Cao	0.544 ± 0.000	0.511 ± 0.005	–	0.523 ± 0.000	0.497 ± 0.027	**0.637 ± 0.002**	0.580 ± 0.004	–
Mammoth	**1.000 ± 0.000**	0.788 ± 0.000	0.697 ± 0.000	**1.000 ± 0.000**	0.739 ± 0.024	0.848 ± 0.000	0.855 ± 0.023	0.582 ± 0.012
MNIST	0.600 ± 0.000	0.713 ± 0.016	**0.767 ± 0.000**	0.733 ± 0.000	0.640 ± 0.053	0.667 ± 0.000	0.653 ± 0.027	0.667 ± 0.000
Hierarchical	**0.535 ± 0.000**	0.278 ± 0.002	0.511 ± 0.002	0.492 ± 0.001	0.083 ± 0.012	0.310 ± 0.009	0.407 ± 0.006	0.072 ± 0.008

Supplementary Note 9 Notes on Hyper-parameter Sensitivity

The most common hyper-parameter of DR methods supposedly controls the balance between local structure and global structure preservation (e.g., t-SNE's perplexity). However, as pointed out by Wang et al.[19], such parameters generally consider the number of nearest neighbors to be preserved when optimizing the low dimensional layout. By that logic, these parameters cannot control global structure since the number of neighbors is a local quantity. Changing this parameter would not preserve global structure, it would only preserve slightly longer-distance local structure. By contrast, PaCMAP does not capture global structure by increasing the number of nearest neighbors. Instead, it applies forces to "mid-near" points, which robustly preserves global structure, and thus bypasses the hyper-parameter tuning problem.

Although manual parameter tuning is undesirable in unsupervised dimension reduction tasks, we note that it is often beneficial to adjust algorithms' parameters according to the size of a dataset in an automated way. DR in larger datasets often benefits when more graph components (e.g., pairs, or triples) are used to optimize the low dimensional embedding. Thus, some algorithms (e.g., PaCMAP) enable the choice of parameters to be adapted to the size of the dataset.

Supplementary Table 9: Centroid distance correlation. We choose k using the same rule for k-nearest classes preservation. Mean and standard deviation are computed over 5 repetitions. **Bold** is used for the best performance, *italics* is used when the result is not significantly different from the best one (defined by a one-sided t-test with P value of 0.05). PCA, ForceAtlas2 and TriMap achieve the best results on this metric.

Dataset	PCA	t-SNE	art-SNE	ForceAtlas2	UMAP	TriMap	PaCMAP	PHATE
Duo 4Eq	**0.976** ± **0.000**	0.928 ± 0.000	0.831 ± 0.000	0.928 ± 0.000	0.508 ± 0.115	0.817 ± 0.047	0.827 ± 0.099	0.754 ± 0.024
Duo 8Eq	0.968 ± 0.000	0.833 ± 0.004	0.799 ± 0.006	**0.983** ± **0.000**	*0.917* ± *0.074*	0.978 ± 0.002	0.920 ± 0.013	0.911 ± 0.013
Kazer	0.900 ± 0.000	0.803 ± 0.011	0.534 ± 0.027	0.822 ± 0.000	0.679 ± 0.023	**0.964** ± **0.002**	0.820 ± 0.019	0.716 ± 0.012
Muraro	0.862 ± 0.000	0.347 ± 0.012	0.545 ± 0.012	0.841 ± 0.000	0.708 ± 0.036	0.755 ± 0.012	0.569 ± 0.126	**0.870** ± **0.006**
Kang	0.560 ± 0.000	0.366 ± 0.010	0.353 ± 0.016	0.579 ± 0.001	0.435 ± 0.017	**0.806** ± **0.015**	0.564 ± 0.017	0.537 ± 0.010
Stuart	**0.843** ± **0.000**	0.564 ± 0.004	0.363 ± 0.021	0.734 ± 0.001	0.267 ± 0.022	0.756 ± 0.006	0.394 ± 0.005	0.478 ± 0.016
Zheng Mouse	**0.745** ± **0.000**	0.322 ± 0.003	–	0.426 ± 0.000	0.450 ± 0.014	0.677 ± 0.005	0.397 ± 0.002	–
Cao	0.400 ± 0.000	0.318 ± 0.005	–	0.303 ± 0.000	0.279 ± 0.051	**0.754** ± **0.001**	0.422 ± 0.007	–
Mammoth	**0.999** ± **0.000**	0.830 ± 0.004	0.859 ± 0.002	0.998 ± 0.000	0.830 ± 0.007	0.867 ± 0.004	0.928 ± 0.004	0.346 ± 0.025
MNIST	**0.864** ± **0.000**	0.770 ± 0.002	0.804 ± 0.002	0.857 ± 0.000	0.786 ± 0.023	0.844 ± 0.001	0.754 ± 0.029	0.799 ± 0.013
Hierarchical	**0.892** ± **0.000**	0.526 ± 0.004	0.636 ± 0.006	0.856 ± 0.000	0.008 ± 0.027	0.413 ± 0.004	0.802 ± 0.002	0.014 ± 0.005

Supplementary References

[1] Romain Lopez, Jeffrey Regier, Michael B Cole, Michael I Jordan, and Nir Yosef. Deep generative modeling for single-cell transcriptomics. Nature Methods, 15(12):1053–1058, 2018.

[2] Jiarui Ding, Anne Condon, and Sohrab P Shah. Interpretable dimensionality reduction of single cell transcriptome data with deep generative models. Nature Communications, 9(1):1–13, 2018.

[3] Junyue Cao, Malte Spielmann, Xiaojie Qiu, Xingfan Huang, Daniel M Ibrahim, Andrew J Hill, Fan Zhang, Stefan Mundlos, Lena Christiansen, Frank J Steemers, et al. The single-cell transcriptional landscape of mammalian organogenesis. Nature, 566(7745):496–502, 2019.

[4] Jonathan S Packer, Qin Zhu, Chau Huynh, Priya Sivaramakrishnan, Elicia Preston, Hannah Dueck, Derek Stefanik, Kai Tan, Cole Trapnell, Junhyong Kim, et al. A lineage-resolved molecular atlas of c. elegans embryogenesis at single-cell resolution. Science, 365(6459), 2019.

[5] Etienne Becht, Leland McInnes, John Healy, Charles-Antoine Dutertre, Immanuel WH Kwok, Lai Guan Ng, Florent Ginhoux, and Evan W Newell. Dimensionality reduction for visualizing single-cell data using umap. Nature Biotechnology, 37(1):38–44, 2019.

[6] Robert A Amezquita, Aaron TL Lun, Etienne Becht, Vince J Carey, Lindsay N Carpp, Ludwig Geistlinger, Federico Marini, Kevin Rue-Albrecht, Davide Risso, Charlotte Soneson, et al. Orchestrating single-cell analysis with bioconductor. Nature Methods, 17(2):137–145, 2020.

[7] Yun Lin, Xiaolei Zhu, Zhigao Zheng, Zheng Dou, and Ruolin Zhou. The individual identification method of wireless device based on dimensionality reduction and machine learning. The Journal of Supercomputing, 75(6):3010–3027, 2019.

[8] Fulin Luo, Liangpei Zhang, Bo Du, and Lefei Zhang. Dimensionality reduction with enhanced hybrid-graph discriminant learning for hyperspectral image classification. IEEE Transactions on Geoscience and Remote Sensing, 58(8):5336–5353, 2020.

[9] Jiaqi Mu, Suma Bhat, and Pramod Viswanath. All-but-the-top: Simple and effective postprocessing for word representations. arXiv preprint arXiv:1702.01417, 2017.

[10] Vikas Raunak, Vivek Gupta, and Florian Metze. Effective dimensionality reduction for word embeddings. In Proceedings of the 4th Workshop on Representation Learning for NLP (RepL4NLP-2019), pages 235–243, 2019.

[11] Karl Pearson. On lines and planes of closest fit to systems of points in space. Philosophical Magazine, 2(11):559–572, 1901.

[12] Warren Torgerson. Multidimensional scaling: I theory and method. Psychometrika, 17(4): 401–419, 1952.

[13] Daniel D Lee and H Sebastian Seung. Learning the parts of objects by non-negative matrix factorization. Nature, 401(6755):788–791, 1999.

[14] Joshua B. Tenenbaum, Vin de Silva, and John C. Langford. A global geometric framework for nonlinear dimensionality reduction. Science, 290(5500):2319–2323, 2000.

[15] Sam T. Roweis and Lawrence K. Saul. Nonlinear dimensionality reduction by locally linear embedding. Science, 290(5500):2323–2326, 2000.

[16] Mikhail Belkin and Partha Niyogi. Laplacian eigenmaps and spectral techniques for embedding and clustering. In Advances in Neural Information Processing Systems, volume 14, pages 585–591. MIT Press, 2001.

[17] Laurens van der Maaten and Geoffrey Hinton. Visualizing data using t-SNE. Journal of Machine Learning Research, 9:2579–2605, 2008.

[18] Leland McInnes, John Healy, and James Melville. UMAP: Uniform Manifold Approximation and Projection for Dimension Reduction. arXiv e-prints, art. arXiv:1802.03426, February 2018.

[19] Yingfan Wang, Haiyang Huang, Cynthia Rudin, and Yaron Shaposhnik. Understanding how dimension reduction tools work: An empirical approach to deciphering t-SNE, UMAP, TriMAP, and PaCMAP for data visualization. Journal of Machine Learning Research, 22, 2021.

[20] Laurens van der Maaten. Accelerating t-SNE using tree-based algorithms. Journal of Machine Learning Research, 15:3221–3245, 2014.

[21] George C. Linderman, Manas Rachh, Jeremy G. Hoskins, Stefan Steinerberger, and Yuval Kluger. Fast interpolation-based t-SNE for improved visualization of single-cell rna-seq data. Nature Methods, 16:243–245, 2019.

[22] El-ad David Amir, Kara L Davis, Michelle D Tadmor, Erin F Simonds, Jacob H Levine, Sean C Bendall, Daniel K Shenfeld, Smita Krishnaswamy, Garry P Nolan, and Dana Pe'er. viSNE enables visualization of high dimensional single-cell data and reveals phenotypic heterogeneity of leukemia. Nature Biotechnology, 31(6):545–552, 2013.

[23] Anna C. Belkina, Christopher O. Ciccolella, Rina Anno, Richard Halpert, Josef Spidlen, and Jennifer E. Snyder-Cappione. Automated optimized parameters for t-distributed stochastic neighbor embedding improve visualization and analysis of large datasets. Nature Communications, 10(5415), 2019.

[24] Jan Niklas Böhm, Philipp Berens, and Dmitry Kobak. A Unifying Perspective on Neighbor Embeddings along the Attraction-Repulsion Spectrum. arXiv e-prints, art. arXiv:2007.08902, July 2020.

[25] Mathieu Jacomy, Tommaso Venturini, Sebastien Heymann, and Mathieu Bastian. Forceatlas2, a continuous graph layout algorithm for handy network visualization designed for the gephi software. PLOS ONE, 9(6):1–12, 06 2014. doi: 10.1371/journal.pone.0098679.

[26] Caleb Weinreb, Samuel Wolock, and Allon M Klein. Spring: a kinetic interface for visualizing high dimensional single-cell expression data. Bioinformatics, 34(7):1246–1248, 2018.

[27] Kevin R Moon, David van Dijk, Zheng Wang, Scott Gigante, Daniel B Burkhardt, William S Chen, Kristina Yim, Antonia van den Elzen, Matthew J Hirn, Ronald R Coifman, et al. Visualizing structure and transitions in high-dimensional biological data. Nature Biotechnology, 37 (12):1482–1492, 2019.

[28] Emma Pierson and Christopher Yau. Zifa: Dimensionality reduction for zero-inflated single-cell gene expression analysis. Genome Biology, 16(1):1–10, 2015.

[29] Davide Risso, Fanny Perraudeau, Svetlana Gribkova, Sandrine Dudoit, and Jean-Philippe Vert. A general and flexible method for signal extraction from single-cell rna-seq data. Nature Communications, 9(1):1–17, 2018.

[30] F William Townes, Stephanie C Hicks, Martin J Aryee, and Rafael A Irizarry. Feature selection and dimension reduction for single-cell rna-seq based on a multinomial model. Genome Biology, 20(1):1–16, 2019.

[31] Shiquan Sun, Jiaqiang Zhu, Ying Ma, and Xiang Zhou. Accuracy, robustness and scalability of dimensionality reduction methods for single-cell rna-seq analysis. Genome Biology, 20(1): 1–21, 2019.

[32] Samuel W Kazer, Toby P Aicher, Daniel M Muema, Shaina L Carroll, Jose Ordovas-Montanes, Vincent N Miao, Ang A Tu, Carly GK Ziegler, Sarah K Nyquist, Emily B Wong, et al. Integrated single-cell analysis of multicellular immune dynamics during hyperacute hiv-1 infection. Nature Medicine, 26(4):511–518, 2020.

[33] Hyun Min Kang, Meena Subramaniam, Sasha Targ, Michelle Nguyen, Lenka Maliskova, Elizabeth McCarthy, Eunice Wan, Simon Wong, Lauren Byrnes, Cristina M Lanata, et al. Multiplexed droplet single-cell RNA-sequencing using natural genetic variation. Nature Biotechnology, 36(1):89, 2018.

[34] Tim Stuart, Andrew Butler, Paul Hoffman, Christoph Hafemeister, Efthymia Papalexi, William M Mauck III, Yuhan Hao, Marlon Stoeckius, Peter Smibert, and Rahul Satija. Comprehensive integration of single-cell data. Cell, 177(7):1888–1902, 2019.

[35] Dmitry Kobak and Philipp Berens. The art of using t-SNE for single-cell transcriptomics. Nature Communication, 10:5416, 2019.

[36] Angelo Duò, Mark D Robinson, and Charlotte Soneson. A systematic performance evaluation of clustering methods for single-cell rna-seq data. F1000Research, 7, 2018.

[37] Mauro J Muraro, Gitanjali Dharmadhikari, Dominic Grün, Nathalie Groen, Tim Dielen, Erik Jansen, Leon Van Gurp, Marten A Engelse, Francoise Carlotti, Eelco Jp De Koning, et al. A single-cell transcriptome atlas of the human pancreas. Cell Systems, 3(4):385–394, 2016.

[38] Grace XY Zheng, Jessica M Terry, Phillip Belgrader, Paul Ryvkin, Zachary W Bent, Ryan Wilson, Solongo B Ziraldo, Tobias D Wheeler, Geoff P McDermott, Junjie Zhu, et al. Massively parallel digital transcriptional profiling of single cells. Nature Communications, 8(1):1–12, 2017.

[39] The Smithsonian Institute. Mammuthus primigenius (blumbach). https://3d.si.edu/object/3d/mammuthus-primigenius-blumbach:341c96cd-f967-4540-8ed1-d3fc56d31f12, 2020.

[40] Andy Coenen and Adam Pearce. Understanding UMAP. https://pair-code.github.io/understanding-umap/, 2019.

[41] Yann LeCun, Corinna Cortes, and CJ Burges. MNIST handwritten digit database. ATT Labs [Online]. Available: http://yann. lecun. com/exdb/mnist, 2, 2010.

[42] Yuhan Hao, Stephanie Hao, Erica Andersen-Nissen, William M Mauck III, Shiwei Zheng, Andrew Butler, Maddie J Lee, Aaron J Wilk, Charlotte Darby, Michael Zager, et al. Integrated analysis of multimodal single-cell data. Cell, 2021.

[43] F Alexander Wolf, Philipp Angerer, and Fabian J Theis. Scanpy: large-scale single-cell gene expression data analysis. Genome Biology, 19(1):1–5, 2018.

[44] Aaron TL Lun, Karsten Bach, and John C Marioni. Pooling across cells to normalize single-cell rna sequencing data with many zero counts. Genome Biology, 17(1):1–14, 2016.
