# Supplementary material for: Towards a comprehensive evaluation of dimension reduction methods for transcriptomic data visualization
Source: Commun Biol. 2022 Jul 19;5:719. doi: 10.1038/s42003-022-03628-x (PMC9296444; doi:10.1038/s42003-022-03628-x)
Supplement: Supplementary file 2 — Description of Additional Supplementary Files [file 42003_2022_3628_MOESM2_ESM.pdf]

Description of Additional Supplementary Files

File name: Supplementary Data 1
Description: The source data of each evaluation in the paper.
