# Supplementary figures and images for: Towards a comprehensive evaluation of dimension reduction methods for transcriptomic data visualization
Source: Commun Biol. 2022 Jul 19;5:719. doi: 10.1038/s42003-022-03628-x (PMC9296444; doi:10.1038/s42003-022-03628-x)

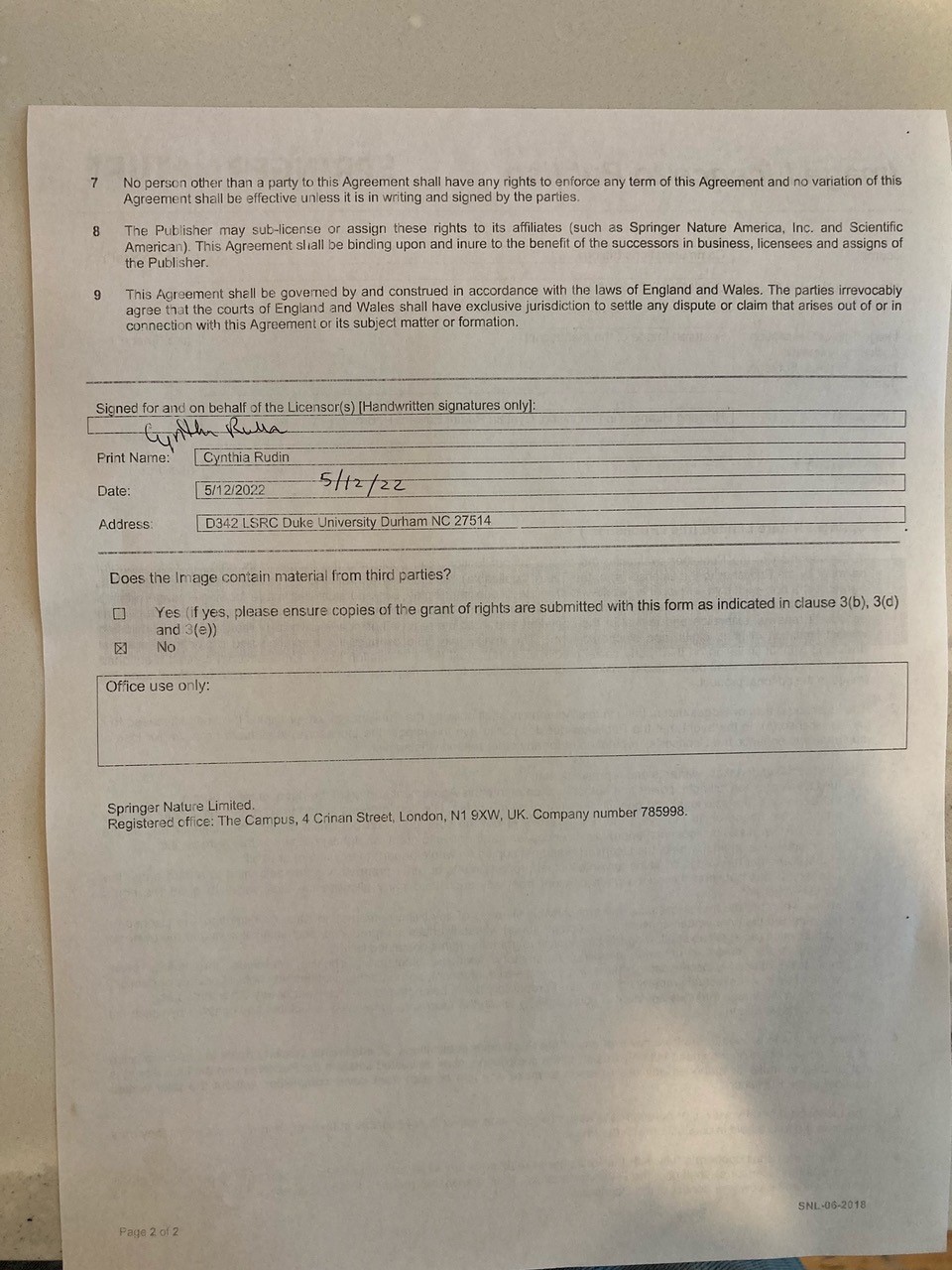

Supplement: Supplementary file 5 — Featured Image License [file 42003_2022_3628_MOESM5_ESM.jpg]

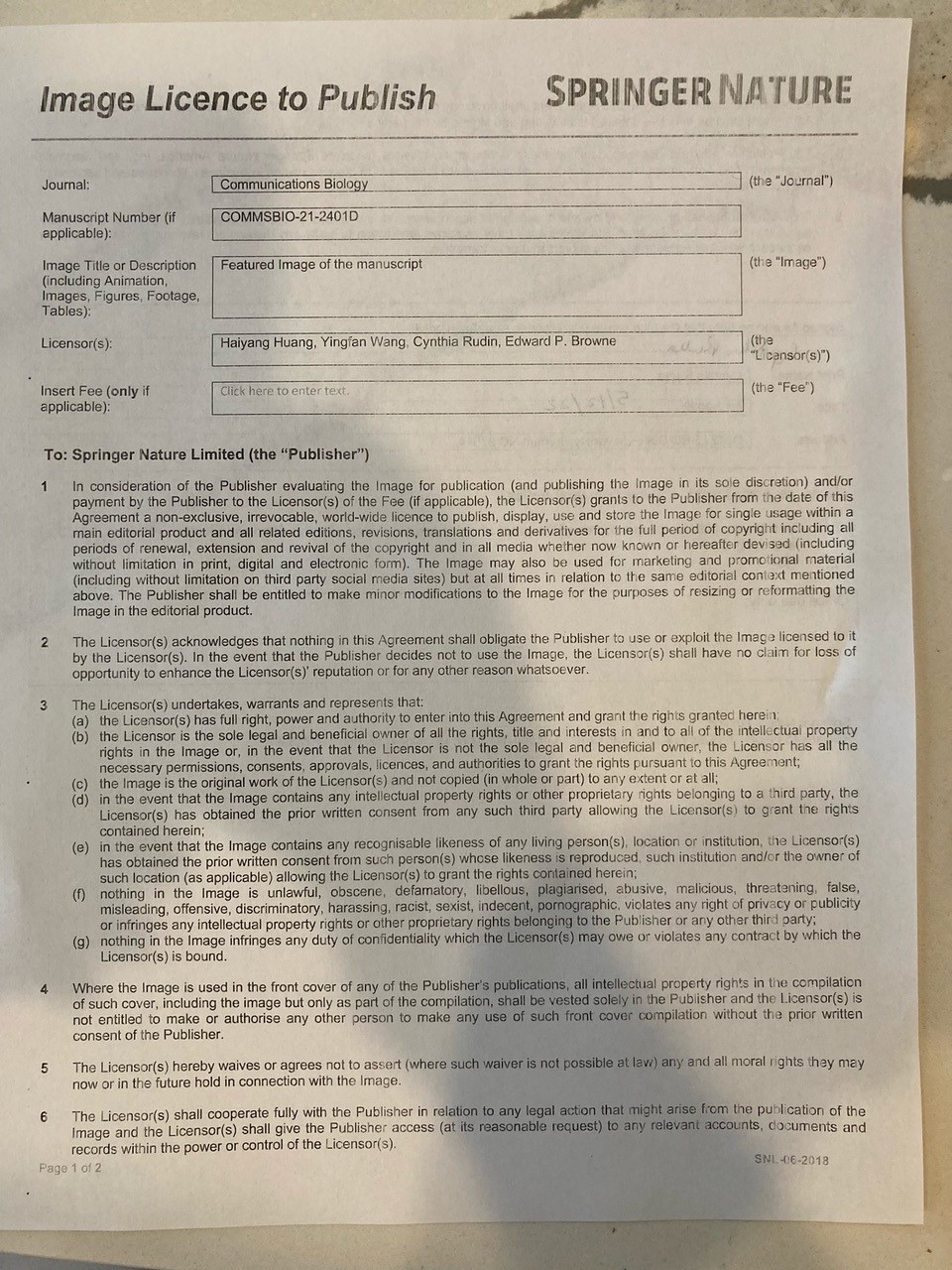

Supplement: Supplementary file 6 — Featured Image License [file 42003_2022_3628_MOESM6_ESM.jpg]
